# Spatial optimization of industrial symbiosis for heat supply of agricultural greenhouses

**DOI:** 10.1111/jiec.13543

**Published:** 2024-08-13

**Authors:** Farzaneh Rezaei, Vanessa Burg, Stephan Pfister, Stefanie Hellweg, Ramin Roshandel

**Affiliations:** 1 Department of Energy Engineering, Sharif University of Technology, Tehran, Iran; 2 Institute of Environmental Engineering, ETH Zürich, Zürich, Switzerland

**Keywords:** agricultural greenhouse, economy of scale, industrial symbiosis, linear programming, optimization, waste heat

## Abstract

Despite the many benefits of greenhouses, it is challenging to meet their heating demand, as greenhouses belong to the most energy-intensive production systems in the agriculture sector. Industrial symbiosis can bring an effective solution by utilizing waste heat from other industries to meet the greenhouse heat demand. This study proposes an optimization framework by which optimum symbiotic relationships can be identified. For this aim, the spatial analysis is integrated into an optimization model, in which geographical, technical, and economic parameters are considered simultaneously to identify the optimal location for developing new agricultural greenhouses. The objective function is to minimize the heating costs, that is, the investment cost of piping and electricity cost for pumping heat-carrying fluid from supplier to demand. The model is applied to the case study of Switzerland, and currently existing municipal solid waste incinerators, cement production plants, and biogas plants are considered potential waste heat sources. Results show that the import of tomato, cucumber, and lettuce to Switzerland can theoretically be replaced by vegetable production in new waste-heat supplied greenhouses (zero import scenarios). Accounting for the economy of scale for pipeline investment costs leads to selecting large-scale greenhouses with a cost reduction of 37%. The optimization results suggest that 10% of the greenhouses needed to satisfy the total domestic demand for lettuce, tomato, and cucumber could be placed on a suitable land plot in the direct vicinity of a waste heat source, with low costs of waste heat supply.

**Supplementary Information:**

The online version of this article (doi:10.1111/jiec.13543) contains supplementary material, which is available to authorized users.

## INTRODUCTION

Demand for greenhouse production rises due to the growing population, the need for cropping season extension, and food security in the case of adverse climatic conditions (Greenhouse Horticulture Market Size & Industry Report, 2023). Greenhouses not only increase the yield by up to ten times, but they can also reduce water consumption by up to 12 times compared with open-field cultivation (Esmaeli & Roshandel, 2020). Nevertheless, the adoption of protected agriculture presents challenges, including substantial investment costs (Fernández et al., 2018), energy consumption (Soussi et al., 2022), and environmental impact (Marcelis et al., 2019). Greenhouse energy demand per unit area can be nearly 100 times that of open field cultivation, varying based on geographical location (Barbosa et al., 2015). Fossil fuel-powered greenhouses strain the energy system and contribute to environmental consequences (Stoessel et al., 2012), such as climate change, for example. Consequently, there is an urgent need to comprehend greenhouse energy systems and investigate practical solutions to minimize energy inputs and associated environmental impacts.

### Greenhouse energy management

Several studies aimed to reduce energy input while maintaining optimal growth conditions. Van Beveren et al. (2015) applied dynamic optimization based on optimum control theory to decrease yearly energy input in a Dutch greenhouse. In addition to temperature and humidity, optimal control strategies of CO_2_ fertilizing (Kläring et al., 2007) and supplementary lighting plans (Pinho et al., 2013) have proven effective in conserving resources and minimizing specific energy (energy per unit yield) in greenhouses.

Academic interest extends to greenhouse design, with studies exploring modern envelopes and technologies (Fabrizio, 2012), suggesting thermal curtains and ground-heated greenhouses (Shukla et al., 2006), proposing movable thermal insulation (Arinze et al., 1986), and considering solar greenhouses as a solution to decrease energy consumption impacts (Tong et al., 2013; Wang et al., 2014).

However, the effectiveness of diverse energy-saving strategies depends on climatic conditions, greenhouse type, growing practices, energy transport, and the embodied energy of greenhouse components. Tailoring solutions to specific locations is crucial for obtaining targeted energy and environmental impact reduction measures, along with yield gains.

### Industrial symbiosis implementation for greenhouses

Another approach to tackle the energy consumption challenge of greenhouse systems is to implement the industrial symbiosis concept, according to which separate entities cooperate in sharing resources and exchanging their waste streams to meet their demands (Chertow, 2000; Fraccascia et al., 2021). For the specific case of greenhouses, the wastes from other industries can be utilized to supply greenhouse needs such as heat, water, CO_2_, or fertilizer (Butturi et al., 2019).

Several studies have investigated the supply of greenhouse heating utilizing waste heat sources. Marton et al. (2010) investigated the industrial symbiosis between a municipal solid waste incinerator (MSWI) and a tomato production greenhouse in Switzerland, where waste heat of the plant condenser is used to heat the greenhouse space. They showed that there are many environmental benefits in this type of symbiotic relationship. In their specific case, 40 kW of the load of the fan of the condenser was sold to the greenhouse, and at the same time, the steam turbine cycle productivity could be raised. Shelford et al. (2016) considered the industrial symbiosis between a greenhouse and an anaerobic digester using livestock manure as input. This study revealed the economic benefits of using biogas plant waste heat for the greenhouse cultivation system. Hence, the co-location of biogas plants and greenhouses can improve waste heat management. Andrews and Pearce (2011) conducted a study of the industrial symbiosis between industrial processes and greenhouses with a technical and economic approach in the northern climate of Canada. They concluded that waste heat from a glass factory would be significantly more economical than the natural gas boiler system for supplying greenhouse heating demand. Yu and Nam (2016) evaluated the feasibility of using the waste heat of power plants for greenhouses in Korea. Their results showed that in Dangjin, Hadong, and Youngdong, 825 ha of greenhouses can be supplied by utilizing about 47% of available energy reserves, and the payback period for all scenarios was 2 to 3 years. In a similar study for Korea, Lee et al. (2016) compared the use of the waste heat potential of MSWIs and power plants for large-scale greenhouses. Hereby, the temperature of hot water, the heat loss potential, and the heat transferring cost to the greenhouse were considered functions of the type of pipe and the distance between the heat source and the greenhouse. Although the investment in long-distance piping increased the payback period, waste heat was the optimal option. Başak and Sevilgen (2016) proposed a technical and economic model for greenhouse heating using waste heat. They evaluated the effect of the indoor temperature of the greenhouse, the type of material for its construction, and the heat transferring cost. The results demonstrated that the impact of indoor temperature on heating costs was significant. Therefore, the type of crop grown in the greenhouse and the specific plant requirements are essential to consider.

### Theoretical background for industrial symbiosis optimization

There are numerous options to establish industrial symbiosis from a set of feasible solutions. Optimization allows to identify the best industrial symbiosis scenario among a range of potential options regarding the chosen objective function and constraints. To provide an overview of the existing literature, we categorized studies investigating the optimization of industrial symbiosis networks in Table Table 1.

**TABLE 1:** A review of the studies about industrial symbiosis optimization.

Papers	Decision variable		Case study	Description	Objective functions
	Location^a^	Technology^b^			
Karlsson and Wolf (2008)			A chemical pulp mill, sawmill, a biofuel upgrading plant, and district heating	A region including industrial plants in Sweden	Min cost
Chae et al. (2010)			A petrochemical plant	A petrochemical complex in South Korea	Min cost
Stijepovic and Linke (2011)		.	Industrial utilities	Imaginary industrial zone	Max profit
Hipólito-Valencia et al. (2014)			Industrial utilities	Three unknown plants containing two hot and cold utilities	Min cost
Gu et al. (2013)			Industrial park	Industrial park—Le Havre in France	Max benefit and max quantity of exchanges
Tashkiri et al. (2015)			Industrial park	Ulsan Eco-Industrial Park in South Korea	Max satisfaction
Zhang et al. (2016)			Industrial park	Industrial park, Jurong Island in Singapore	Min payback period and min emission reduction
Afshari et al. (2018)			Three industrial plants	Industrial zone in France	Min cost and min environmental impact
Nouinou et al. (2019)			An oil refinery, a power plant, and a construction company	–	Max amount of flow exchanges and max total economic gain
Cao et al. (2020)			National (China)	Iron and steel industries, cement industries, thermal power industries, social sector, chemical industries, building material industries, etc. in China	Max energy conservation, min emission reduction, and min investment cost
Afshari et al. (2020)			Three industrial plants	Industrial zone in France	Min cost, min environmental impact, and max social preference
Pang et al. (2023)			A hypothetical industrial park	Utilization of renewable energy technologies (e.g., photovoltaic solar panels and wind turbines) and energy storage technologies (e.g., hydrogen and thermal energy storage) in supplying demand	Min annualized investment and operation cost in addition to carbon tax and min CO_2_ emission
Biox et al. (2023)			Industrial park	Yeosu park in South Korea	Min net present cost (NPC)
This study			National (Switzerland)	Greenhouses supplied by waste heat from biogas plants, municipal solid waste incinerators, and cement production plants in Switzerland	Annualized minimum cost of the industrial symbiosis

*Note*: A red cross means “no consideration,” while a green checkmark means “consideration.”

^a^Geographical characteristics are utilized to determine which location is optimal for establishing industrial symbiosis between supply and demand, as indicated by the decision variable.

^b^The process of optimization involves the identification of suitable technologies (e.g., organic Rankine cycle [ORC]) to facilitate symbiotic supply and demand.

A small number of studies have used optimization models to find the optimal waste heat exchanges at the scale of eco-industrial parks. In Chae et al. (2010), a mixed integer linear programming model is used to optimize a waste heat utilization network between 27 industrial complexes and 15 neighboring communities in a petrochemical complex in South Korea. Almost all of the considered heat demands could be satisfied by waste heat in the given case, which led to economic and environmental benefits (Karlsson & Wolf, 2008). In reference Zhang et al. (2016), single- and multi-objective optimization models are presented to assess the opportunities of waste heat recovery potentials at the park level in Jurong Island, Singapore, comprising five plants and two communities. Three objective functions of energy efficiency of the park, payback time period for the establishment of the waste heat transportation system (pipeline construction), and CO_2_ emission reduction are compared as single- and multi-objective optimization models. Their results highlight the importance of the selected objective function, which affected the optimization solutions. In reference Afshari et al. (2018, 2020), single- and multi-objective mixed integer linear programing models are presented to minimize the total cost and the environmental impact on energy suppliers and users in an industrial park and the residential neighborhoods in France. These articles showed the effectiveness of using the energy of the industrial park as a valuable source of recovered energy for residential buildings. Additionally, they found that the building connections to the main pipelines represented the primary financial barrier. The authors also evaluated the use of organic Rankine cycle (ORC) systems in energy symbiosis networks. Recent research has concentrated on improving existing industrial symbiosis networks. Biox et al. (2023) presented a methodology to optimize the design of flexible networks in industrial symbiosis. To consider network flexibility, the resilience index is proposed, which can be defined as the smallest maximum deviation of input network parameters (e.g., utility demands and cost of equipment) that a system can endure without becoming infeasible. They conclude that although the high flexibility leads to increased adaptability of the network to input parameter fluctuations, the overall cost (capital, operation, expenditures, and resource cost) will be increased. Pang et al. (2023) proposed a multi-objective, multi-period optimization model to optimize the size of components in an industrial symbiosis utilizing a hybrid renewable energy conversion and storage system. Two objective functions are considered: (1) minimization of annualized investment and operation cost in addition to carbon tax and (2) minimization of CO2 emission. The main output of their optimization model is the optimal hourly energy flows (electricity, heat, cooling, and hydrogen) using photovoltaic, wind turbines, fuel cells, ORC, heat pumps, boilers, and chillers.

### Research gaps and study objectives

To the best of the authors’ knowledge, no prior studies have performed location-based optimization of waste-heat use on a large geographical scale spanning beyond industrial parks, considering various technologies. This study aims to develop such a large-scale optimization framework that informs the decision-making of policymakers, waste-heat supplying industry, and greenhouse operators with regard to identifying the most cost-efficient locations for waste heat-supplied greenhouses and promoting a sustainable and efficient agricultural system. The integration of detailed geographical and technical conditions in the optimization of an industrial symbiosis network is novel, as existing studies mostly lack consideration of detailed geographical and technical conditions in the optimization framework (Table Table 1). Moreover, the (national) scale of our study is exceptionally large compared to existing work, which has focused mainly on industrial zones or a limited number of industrial participants. Finally, the application of such a large-scale optimization to the case of greenhouses as demand points for low-temperature heat is new.

In this study, we specifically consider greenhouses as the demand side to show how industrial symbiosis can help the agricultural sector lower resource consumption and costs. For this aim, we utilized a heat transfer model to estimate the agricultural greenhouses’ peak heat demand, which is the input of our optimization model. This enables us to see the effect of changes in greenhouse heat demand on the optimization results and is especially useful when a new practice (e.g., new ventilation control system) or technology (e.g., heat curtain) is considered to be implemented into the industrial symbiosis network. The proposed optimization model not only identifies the most cost-effective locations for new greenhouses but also gives insights on whether or not it is optimal to use industrial waste heat for three types of crop cultivations: cucumber, lettuce, and tomatoes.

Utilizing a waste-heat-to-electricity technology in industrial symbiosis can potentially improve economic and environmental performance due to reducing exergy loss by producing electrical energy. Among the low-temperature waste-to-electricity technologies, ORC has been the most common in recent years (Lecompte et al., 2015; Quoilin et al., 2013) since it offers advantages such as simplicity (Pereira et al., 2018), availability of equipment (Quoilin et al., 2011), flexibility in being used at diverse capacities (Simpson et al., 2019), unmanned operation, and small need of maintenance (Lecompte et al., 2015). This is also important because, recently, the ORC system showed promising results in several practical cases, especially in cement industries (Santarossa, n.d.).

In our study, two pathways for heating greenhouses using waste heat are considered and compared. In the first pathway, the hot fluid (heated by waste heat) is directly transported through pipelines to heat the greenhouses, while in the second pathway, the waste heat is first utilized in the ORC evaporator to generate electricity and the rejected heat from ORC condenser is then used for heating greenhouses.

Our optimization framework also incorporates the effect of economies of scale for capacities (adjustment of investment costs for various capacity scales of piping and ORC systems) to produce more realistic and pertinent outcomes. The developed methodology is not restricted to greenhouses but may be applied as an optimization framework to prioritize the symbiotic potentials between any potential supply source and demand.

## METHODS

**FIGURE 1:**
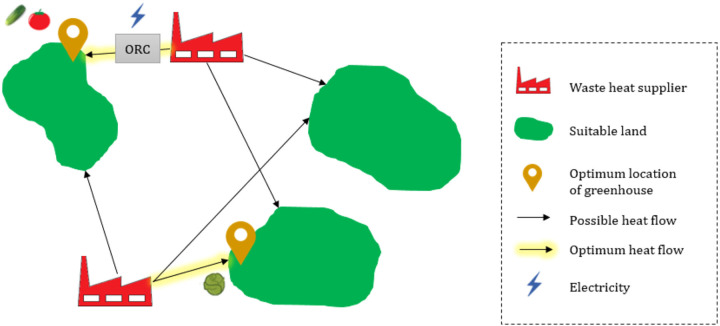
Possible symbiotic pathways in a hypothetical region.

Waste heat sources and suitable lands for agricultural greenhouses were characterized as input to the optimization, which performs the matching (Figure 1). Each point or area includes specific attributes such as the available cultivation area, waste heat potential, energy demand, and distances to other points. Therefore, geographic, technical, and economic parameters are considered simultaneously as inputs of the optimization framework to prioritize the optimal pathway for developing greenhouses utilizing a clean energy strategy. Greenhouse area and its location are the decision variables of this optimization framework, and diverse opportunities of waste heat are evaluated together as available resources.

**FIGURE 2:**
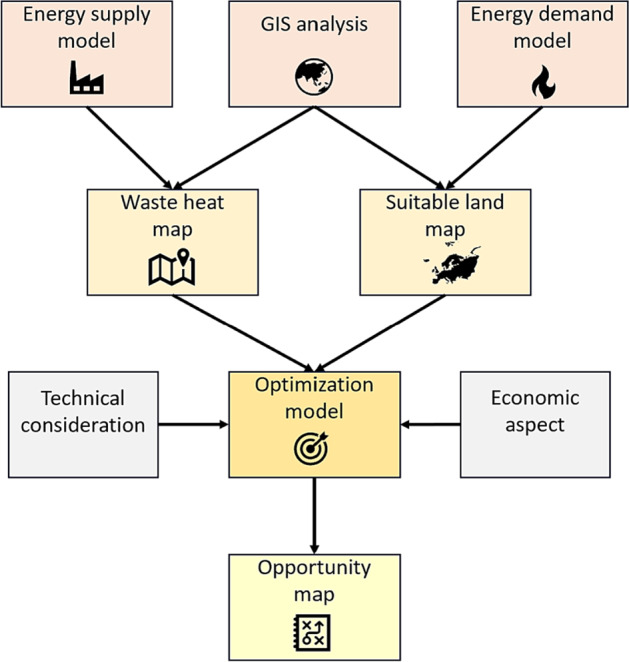
Proposed workflow. GIS, geographical information system.

Figure 2 displays the workflow proposed in this study. The method applied is categorized into the following parts: The first step includes spatial analysis, energy supply model, and greenhouse energy demand model. Spatial analysis is the organized elimination of regions that do not fall into the specified category of land suitable for greenhouse development including artificial surfaces, forests and semi-natural areas, wetlands, and water bodies. The use of the Copernicus database (Copernicus, Global Monitoring for Environment & Security, the European Union's Earth observation programme| Copernicus, n.d.) serves as the primary data source for the geographical information system (GIS) in this process. Regarding the energy supply model, information about target industries, including biogas plants, MSWIs, and cement production plants, is collected (more explanation in Section 2.2.1), and waste heat for each supplying plant is calculated. Climate data (including radiation and temperature on an hourly basis) are required to estimate the greenhouse energy model, which was extracted from (Hans Ertel Centre for Weather Research) for the last 20 years for all sites with suitable land areas for greenhouses. The maximum projected greenhouse heating energy demand for each suitable land is estimated by identifying the coldest hour and calculating the maximum heating demand throughout each year for each suitable land. Then, the average maximum heating demand of these 20 years is assigned to related suitable land. We calculated heat demand on a 1 × 1 km^2^ spatial resolution. The second step is to visualize and present: (1) the potential waste heat map based on the results of the energy supply model (waste heat potential) and (2) the suitable land map with the related greenhouse energy demand. The final step is that all obtained data are utilized as input to the superstructure optimization that provides an opportunity map (the output of optimization framework, which displays optimal pathways for conducting industrial symbiosis relationship) by considering technological and economic aspects.

### Optimization framework

#### Formulation

In the proposed optimization problem, the main decision variable is the greenhouse area (${{X}_{i,j,l,k}}$) that includes indices $i,\,j,\,k$, and $l$ that denote waste heat sources, suitable land, technological pathway (with or without ORC), and crop type (consisting of tomato, cucumber, and lettuce), respectively.

The objective function of the optimization model is the minimum total annual cost, including investment and operation costs of piping and investment and operation costs of waste-to-electricity technology (ORC), as well as the profit of selling electricity to the grid (in the case of the ORC technology). Note that corresponding costs for constructing new greenhouses are neglected here since this is the same for all options and does not affect the optimization. The objective function formulation presented in Equation (1) includes the sum of several types of costs, referring to 1 year (investment costs are annualized) and income.

1 $${\mathrm{Min\ total\ annual\ cost}} \;=\; \sum_{i = 1}^I \sum_{j = 1}^J \sum_{n = 1}^N \ {\mathrm{IN}}{{{\mathrm{V}}}_n} \times t_{i,j}^n \times {{d}_{i,j}} \times {\mathrm{CRF}} + \sum_{k = 1}^K \sum_{i = 1}^I \sum_{j = 1}^J \sum_{l = 1}^L {{C}_{{\mathrm{OP}}}} \times {{d}_{i,j}} \times {{X}_{i,j,l,k}} \times {\mathrm{P}}{{{\mathrm{D}}}_{l,j}}\ \times {\mathrm{C}}{{{\mathrm{F}}}_1} + \sum_{i = 1}^I \sum_{j = 1}^J \sum_{m = 1}^M {\mathrm{SC}}_{{\mathrm{ORC}}}^m \times {\mathrm{CA}}{{{\mathrm{P}}}_{{\mathrm{orc}}}}_{i,j}^m \times {\mathrm{CRF}}\nonumber\\ \;\; + \sum_{i = 1}^I \sum_{j = 1}^J \sum_{m = 1}^M {\mathrm{OM}}_{{\mathrm{ORC}}}^m \times {\mathrm{CA}}{{{\mathrm{P}}}_{{\mathrm{orc}}}}_{i,j}^m \times {\mathrm{C}}{{{\mathrm{F}}}_2}\ - \ \sum_{m = 1}^M \sum_{i = 1}^I \sum_{j = 1}^J {\mathrm{P}}{{{\mathrm{r}}}_{{\mathrm{el}}}} \times {\mathrm{CA}}{{{\mathrm{P}}}_{{\mathrm{orc}}}}_{i,j}^m \times {\mathrm{C}}{{{\mathrm{F}}}_2}$$

Term 1 is the investment cost of piping where ${\mathrm{IN}}{{{\mathrm{V}}}_n},\ t_{i,j}^n,\ {{d}_{i,j}}$ and ${\mathrm{CRF}}$ denote the investment cost of heat pipeline in discretized section $n$ (CHF/km), binary variable according to the discretized sections ($n$) for heat pipeline investment cost between the supplier $i$ and the land $j$, distance between source $i$ and demand $j$ (km), and capital recovery factor, respectively. Term 2 represents the operational cost of pump power for transferring heat where ${{C}_{{\mathrm{OP}}}},\ \ {\mathrm{P}}{{{\mathrm{D}}}_{l,j}}$, and ${\mathrm{C}}{{{\mathrm{F}}}_1}$ denote operation cost coefficient (CHF/km MWh), greenhouse heating demand for the possible greenhouse which could be located in land $j$ for crop $l$ (MW/ha), and hours that the greenhouse needs heating (h), respectively. Term 3 denotes the investment cost of waste heat to electricity technology (ORC), where ${\mathrm{SC}}_{{\mathrm{ORC}}}^m$ and ${\mathrm{CA}}{{{\mathrm{P}}}_{{\mathrm{orc}}}}_{i,j}^m$ are the investment costs of ORC technology in discretized section $m$ (CHF/MW) and capacity of ORC in pathway $i,j$ in the discretized section $m$ (MW). Term 4 is the operation and maintenance cost of the ORC system where ${\mathrm{OM}}_{{\mathrm{ORC}}}^m$ and ${\mathrm{C}}{{{\mathrm{F}}}_2}$ are the maintenance cost coefficient for ORC (CHF/MWh) and capacity factor of ORC technology (h). Finally, the last term is the income from the sale of electricity generated from ORC to the grid, where ${\mathrm{P}}{{{\mathrm{r}}}_{{\mathrm{el}}}}$ denotes the electricity selling price (CHF/MWh). To account for the impact of the economy of scale on investment cost, the parameters *n* and *m* are employed to denote the discretized sections utilized for the heat pipeline investment cost *ϵ* {1,…, *N*} and the ORC investment cost *ϵ* {1,…, *M*}, respectively. Section 1 of the supplementary information provides further elaboration on the effect of the economy of scale on investment cost.

The capital recovery factor is calculated using Equation (2) where $r$ and $y$ represent the discount rate and number of annuities, respectively:

2 $${\mathrm{CRF\ }} = \frac{{r{{{\left( {1 + r} \right)}}^y}}}{{{{{\left( {1 + r} \right)}}^y} - 1}}\ $$

The constraints for potentials of waste heat and suitable land are described in Equations (3)–(5). Equation (3) is defined to ensure that related greenhouse areas are satisfied for all vegetables, including tomatoes, cucumbers, and lettuce, where ${\mathrm{T}}{{{\mathrm{A}}}_l}$ denotes annual demand for each vegetable type $l$ (ha). Equation (4) guarantees that the total area of greenhouses does not exceed the suitable land area in a specific location where ${{A}_j}$ denotes the available suitable area in the land *j* (ha). Equation (5) requires that the peak heat demand must be lower or equal to industries’ total waste heat potential in which ${\mathrm{hlc}}$ and ${{E}_i}$ are heat loss coefficients coming from heat transfers (%/km) and waste heat potential of the supplier $i$ (MW), respectively.

3 $$\sum_{i = 1}^I \sum_{j = 1}^J \sum_{k = 1}^K {{X}_{i,j,l,k}} \ge {\mathrm{T}}{{{\mathrm{A}}}_l}$$

4 $$\sum_{i = 1}^I \sum_{l = 1}^L \sum_{k = 1}^K {{X}_{i,j,l,k}} \le {{A}_j}$$

5 $$\sum_{j = 1}^J \sum_{l = 1}^L \sum_{k = 1}^K {{X}_{i,j,l,k}} \times {\mathrm{P}}{{{\mathrm{D}}}_{l,j}} \times \left( {1 + {\mathrm{hlc}} \times {{d}_{i,j}}} \right) \le {{E}_i}$$

Equations (6)–(11) affect the economy of scale of the investment cost of pipelines and ORC. Economy-of-scale effects occur when a larger investment leads to a smaller cost per unit of output (Hanson, 1964). This would require nonlinear terms in the objective function, which would increase the complexity of the model. To adjust the investment cost while keeping the optimization as a linear problem, the linearization method was applied. Utilizing binary variables, it is possible to discretize the pipeline investment cost in the objective function into specific ranges corresponding to related pipeline capacity (heat power) (Boyd, 2004). The comprehensive explanation of the economy of scale is presented in Supporting Information S1,Appendix SI-1.

Equation (6) identifies the heating capacity ($\sum_{n = 1}^N {{a}_n}t_{i,j}^n$) for the selected pathway according to the range of heating carried by the pipeline for an optimum greenhouse, and Equation (7) means that only one pathway can be selected. Equation (8) determines what range of ORC capacity ($\sum_{m = 1}^M {{b}_m}z_{i,j}^m$) can be selected for the optimum pathway, where ${\mathrm{rati}}{{{\mathrm{o}}}_{{\mathrm{HE}}}}$ denotes the heat-to-electricity ratio in an ORC system. Equation (9) restricts the model to choose one specific ORC capacity for the optimum pathway. Equation (10) and (11) assign the suitable ORC capacity according to the heat demand needed for the greenhouse.

6 $$\sum_{l = 1}^L {{X}_{i,j,l,1}} \times {\mathrm{P}}{{{\mathrm{D}}}_{l,j}} \le \sum_{n = 1}^N {{a}_n}t_{i,j}^n\ \quad n = 1, \ldots ,\ N\ $$

7 $$\sum_{n = 1}^N \ t_{i,j}^n = 1\ t_{i,j}^n\epsilon \ \left\{ {0,1} \right\}\ $$

8 $$\ \frac{{\sum_{l = 1}^L {{X}_{i,j,l,2}} \times {\mathrm{P}}{{{\mathrm{D}}}_{l,j}}}}{{{\mathrm{rati}}{{{\mathrm{o}}}_{{\mathrm{HE}}}}\ }} = \sum_{m = 1}^M {{b}_m}z_{i,j}^m{\mathrm{\ \ }} \quad m = 1, \ldots ,\ M\ $$

9 $$\ \sum_{m = 1}^M z_{i,j}^m = 1\ z_{i,j}^m\epsilon \ \left\{ {0,1} \right\}$$

10 $${\mathrm{CA}}{{{\mathrm{P}}}_{{\mathrm{orc}}}}_{i,j}^m \le \frac{{\sum_{l = 1}^L \left( {{{X}_{i,j,l,2}} \times {\mathrm{P}}{{{\mathrm{D}}}_{l,j}}} \right)}}{{{\mathrm{rati}}{{{\mathrm{o}}}_{{\mathrm{HE}}}}\ }}$$

11 $${\mathrm{CA}}{{{\mathrm{P}}}_{{\mathrm{orc}}}}_{i,j}^m \ge \frac{{\sum_{l = 1}^L \left( {{{X}_{i,j,l,2}} \times {\mathrm{P}}{{{\mathrm{D}}}_{l,j}}} \right)}}{{{\mathrm{rati}}{{{\mathrm{o}}}_{{\mathrm{HE}}}}\ }} - \left( {1 - z_{i,j}^m} \right) \times {\mathrm{Big\ }}M$$

#### Greenhouse heat demand

This study addresses the Venlo greenhouse, which features a glass cover structure with a total heat loss coefficient (U-value) of 4 ${\mathrm{W}}/{{{\mathrm{m}}}^2}/{\mathrm{K}}$ and an average ventilation rate of 2.1 ×10^−4^$1/{\mathrm{s}}$. The aforementioned greenhouse design is widely prevalent in European nations (Burg et al., 2020). The inside temperatures of tomato, cucumber, and lettuce greenhouses are recorded at 20°C, 23°C, and 17°C, respectively (Esmaeli & Roshandel, 2020). Further details can be found in the Supplementary Material.

${\mathrm{P}}{{{\mathrm{D}}}_{l,j}}$ denotes the peak greenhouse heat demand for one greenhouse unit (e.g., hectare) regarding the corresponding longitude and latitude. To calculate ${\mathrm{P}}{{{\mathrm{D}}}_{l,j}}$, the energy balance of the greenhouse (Equation (12)) should be calculated to obtain the peak heat power on the coldest day of the year, which is also affected by geographical conditions and crop type (Andrews & Pearce, 2011).

12 $$ \def\eqcellsep{\;}\begin{array}{@{}*{1}{l}@{}} {{\mathrm{P}}{{{\mathrm{D}}}_{l,j}} = {\mathrm{\ Max}}\left( {\ {{Q}_{{\mathrm{conv}}}}\ \left( {l,j,\ t} \right) + {{Q}_{{\mathrm{vent}}}}\ \left( {l,j,\ t} \right) - \ {{Q}_{{\mathrm{solar}}}}\ \left( {l,j,\ t} \right)} \right)}\\ {t = 1,\ 2,\ \ldots ,\ 8760} \end{array} $$

where $l,j,\ t$ denote crop type, location, and hour of the year. ${{Q}_{{\mathrm{solar}}}}$ is the energy flux coming from solar radiation, ${{Q}_{{\mathrm{vent}}}}$ corresponds to the heat transfer due to ventilation, and ${{Q}_{{\mathrm{conv}}}}$ is the heat transfer from covering. This equation can be simplified and presented as Equation (13), neglecting solar heat gains (Andrews & Pearce, 2011).

13 $$ \def\eqcellsep{\;}\begin{array}{@{}*{1}{l}@{}} {{\mathrm{P}}{{{\mathrm{D}}}_{l,j}} = {\mathrm{\ Max}}\left( {{\mathrm{UA\ }}\left( {{{T}_i}\ \left( l \right) - \ {{T}_o}\ \left( {j,\ t} \right)} \right) + {{C}_{{\mathrm{air}}}}\varphi {{\rho }_{{\mathrm{air}}}}\ ({{T}_i}\ \left( l \right) - \ {{T}_o}\ \left( {j,\ t} \right)\ } \right))}\\ {t = 1,\ 2,\ \ldots ,\ 8760} \end{array} $$

where $A$ is the surface area of the greenhouse (${{{\mathrm{m}}}^2}$), $U$ is the total heat loss coefficient (${\mathrm{W/}}{{{\mathrm{m}}}^{\mathrm{2}}}{\mathrm{K}}$), ${{C}_{{\mathrm{air}}}}$ is the specific heat capacity of the air (${\mathrm{J/kgK}}$), *φ* is the ventilation rate (${{{\mathrm{m}}}^3}{\mathrm{/s}}$), ${{\rho }_{{\mathrm{air}}\ }}$ is the air density (${\mathrm{kg/}}{{{\mathrm{m}}}^{\mathrm{3}}}$). ${{T}_i}$ and ${{T}_o}$ are the inside and outside temperature of the greenhouse (${\mathrm{K}}$), respectively.

### Case study

In Switzerland, retailers wish to replace the import of vegetables (including lettuce, cucumber, and tomato) with local food production (Burg et al., 2020). Therefore, we focus on Switzerland as a case study. In this study, we assess if and at which costs the imported aforementioned crops could be replaced with local food produced with clean energy (utilizing waste heat to meet energy demand). We also determine optimal greenhouse locations, waste heat sources, utilized technology, and crop type.

#### Waste heat suppliers

In this study, three waste heat sources are selected as suppliers: cement production plants, MSWIs, and biogas plants from the agricultural sector. Cement production plants with a specific energy consumption of 3.94 Gj/tonne clinker (Zuberi & Patel, 2017) are particularly interesting since this industry is regarded as one of the top three energy-intensive ones, contributing 9% of the industry's final energy demand in Switzerland (Guerra & Kast, 2015). MSWIs, which are mainly close to urban areas, include low-quality waste heat potential in the hot stream of the steam turbine cycle condenser (about 55°C). This waste heat cannot be utilized for many other purposes because of its low-temperature range (Marton et al., 2010), but it is still appropriate for heating agricultural greenhouses. Hence, MSWIs also have particular importance. We know from previous studies (Quoilin et al., 2013) that the potential of biogas plants is substantial. For example, it was estimated that up to 1500 agricultural biogas facilities could be necessary to valorize the potential of manure alone. In addition, agricultural biogas plants have the advantage of being located in agricultural areas and operated by farmers. Biogas plants are also considered because they can provide other potentials for industrial symbiosis, like CO_2_ and digestate, in addition to heat pathways (Baştabak & Koçar, 2020; Leitfaden Abwärmenutzung auf Biogasanlagen, n.d.; Patricio et al., 2017); therefore, their connection with greenhouses can generate many benefits. Data availability is also another reason for choosing these waste heat sources.

##### Cement production plants

Switzerland has six cement production plants with a total clinker production of 5 million tonnes (Zuberi & Patel, 2017). The main waste heat flows belong to kiln exhaust and cooler (CORDIS, cordis.europa.eu, 2022), but these flows are utilized in many plants for preheating the raw materials. These waste heat flows have low temperatures but are still suitable for greenhouse applications. Practical waste heat potential (${{E}_c}$) can roughly be estimated by Equation (14):

14 $$\ {{E}_c} = {{\eta }_c}\ \times \left( {\alpha + \beta } \right) \times C{{a}_c} \times {\mathrm{SEC}}$$

where ${{\eta }_c}$ is the practical heat recovery factor, $\alpha$ is the stack loss, $\beta$ is the cooler loss, ${\mathrm{C}}{{{\mathrm{a}}}_c}$ is the clinker production of each site, and ${\mathrm{SEC}}$ is the specific energy consumption. It should be noted that in Equation (14), all coefficients, except for the clinker capacity (${\mathrm{C}}{{{\mathrm{a}}}_c}$), were assumed to be the same for all cement production plants in Switzerland. However, cement production plants’ age and used technology may affect the heat recovery characteristics.

##### Municipal solid waste incinerators

There are 30 MSWIs in Switzerland, generating 1.8 million MWh of electricity annually and 3.9 million MWh of useful heat for industry and district heating (Einheitliche Heizwert- und Energiekennzahlenberechnung der Schweizer KVA nach europäischem Standardverfahren, 2020). However, there is still a so-far unused waste heat potential in hot water steam from the condenser of the steam turbine cycle with a low temperature (Marton et al., 2010) that could be used for agricultural greenhouses. According to an MSWI report published by the Swiss Federal Office of Energy (Einheitliche Heizwert- und Energiekennzahlenberechnung der Schweizer KVA nach europäischem Standardverfahren, 2020), where the available energy balance table is presented, the condenser waste heat potential in an MSWI can be calculated based on Equation (15).

15 $$\ {{E}_{{\mathrm{in}}}} = {{\eta }_i}\ \times \left( {{\mathrm{SW}} \times {{e}_b} - {\mathrm{CH}} - {\mathrm{SC}} - {\mathrm{EL}} - {\mathrm{SE}}} \right)$$

where ${{E}_{{\mathrm{in}}}}$ is the waste heat potential of the incineration plant, ${{\eta }_i}$ the heat recovery factor, ${\mathrm{SW}}$ is the solid waste entering the boiler, ${{e}_b}$ the boiler efficiency, ${\mathrm{CH}}$ is utilized as district heating, ${\mathrm{SC}}$ is the heat self-consumption, ${\mathrm{EL}}$ is the electricity sold to grid, and ${\mathrm{SE}}$ the electricity self-consumption.

##### Biogas plants

Switzerland has 153 agricultural and industrial biogas plants utilizing combined heat and power (CHP) to generate electricity and heat (Dokumentation Geodatenmodell, Biogasanlagen, 2022). A significant part of the produced heat is needed for heating the fermenter surface, with an average heat demand of 45% of the produced heat and a maximum of about 60% in the cold season (C, 2021). Swiss biogas plants have so far been supported mainly by an electricity-based subsidy scheme (feed-in tariff or one-time remuneration). Hence, less attention was given to heat utilization, which was mainly regarded as a by-product (Scholwin & Nelles, 2013). However, to increase the overall energy (and economic) efficiency, the heat from the CHP units should be utilized as much as possible. Many biogas plants have found solutions to use at least part of the available heat, for example, by heating farms or nearby buildings or for drying purposes. However, even though the utilization of waste heat from biogas plants has steadily increased in the past, a considerable potential is still untapped (Burg et al., 2019; Scholwin & Nelles, 2013; Stürmer et al., 2021; Weinand et al., 2019). In this regard, using waste heat in agricultural greenhouses seems a promising approach. The waste heat potential of biogas plants can roughly be obtained by Equation (16):

16 $$\ {{E}_b} = {{\tau }_{H/E}}\ \times {\mathrm{EB}} \times \varphi $$

where ${{E}_b}$ is the waste heat potential of biogas plants, ${{\tau }_{H/E}}$ the ratio of heat recovery to electricity, ${\mathrm{EB}}$ the electricity generated, and $\varphi$ the percentage of heat not used for the fermenter. The complete list of the optimization model coefficients and their values for selected sources are presented in Supporting Information S1,Appendix SI-2.

## RESULTS

### Waste heat supplier map for developing greenhouses

**FIGURE 3:**
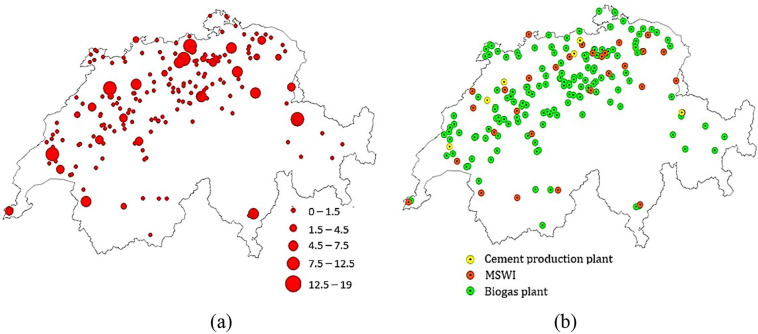
Selected waste heat source suppliers: (a) locations and (b) practical waste heat potential map (MW) of cement production plants, municipal solid waste incinerators (MSWIs), and biogas plants in Switzerland. The underlying data can be found in Supporting Information S2, Appendix SI-4.

Figure 3 shows the location of selected waste heat suppliers (biogas plants, MSWIs, and cement production plants) and the practical waste heat potential for the aforementioned sources. Suppliers with larger practical waste heat potential are mostly placed in the northern and western parts of the case study region. The greater hotspots correspond to MSWIs and cement production plants, and smaller hotspots that are sparsely located throughout the country are biogas plants.

### Heating demand for greenhouse systems

**FIGURE 4:**
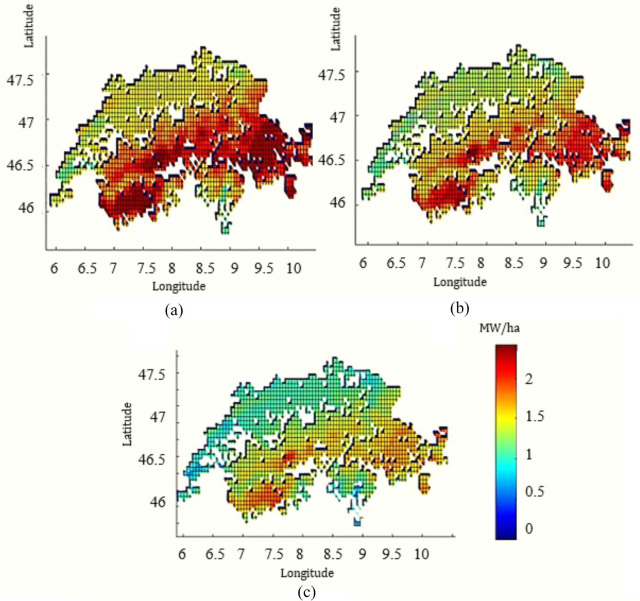
Peak heat demand for suitable lands (MW/ha) for (a) tomato, (b) cucumber, and (c) lettuce greenhouses in Switzerland. The underlying data can be found in Supporting Information S2, Appendix SI-5.

Figure 4 indicates how the geographical location affects the peak heat demand of greenhouse systems. In addition to geographical location, crop type is impactful; for example, tomato production requires a higher indoor greenhouse temperature than lettuce. Taking both factors (geography and crop type) into account, peak heat demand varies from 0.5 to 2 MW/ha.

### Optimization results

The outputs of Sections 3.1 and 3.2 and the input parameters (available in Supporting Information S1,Appendix SI-2) are used as coefficients of the optimization framework. The only parameter that remains to be determined is the total greenhouse area (for lettuce, tomato, and cucumber) that should be targeted (${\mathrm{T}}{{{\mathrm{A}}}_l}$) in Equation (3). As mentioned before, the goal defined in this study is to replace imported vegetables (tomato, cucumber, and lettuce) by establishing new greenhouses with minimal cost.

The main input of the optimization framework is the targeted greenhouse area, which depends on vegetable yield and demand. It is worth mentioning that there are ranges for yield of selected crops; 56.5–70 kg/m^2^ year for tomato (Heuvelink et al., 2006; Torrellas et al., 2012), 65−76 and 90−147 kg/m^2^ year for cucumber (Heuvelink et al., 2006; Kaukoranta et al., 2014), and 35−41.5 kg/m^2^ year for lettuce (Barbosa et al., 2015). According to Statistische Erhebungen und Schätzungen über Landwirtschaft und Ernährung, Statistiques et évaluations concernant l'agriculture et l'alimentation (SES report) (2021), 43,129, 21,855, and 36,992 tonne/year vegetable imports are considered for cucumber, tomato, and lettuce, respectively. The yield of 75 kg/m^2^ year for cucumber, 60 kg/m^2^ year for tomato, and 40 kg/m^2^ year for lettuce gives a greenhouse area demand of 30, 72, and 93 ha for cucumber, tomato, and lettuce, respectively.

In Switzerland, the greenhouse area with solid foundations reached 471 ha by 2019 (Swiss greenhouse area is growing, 2019), meaning (195 ha) 40% more greenhouse area is needed for local food production of cucumbers, tomatoes, and lettuce.

**FIGURE 5:**
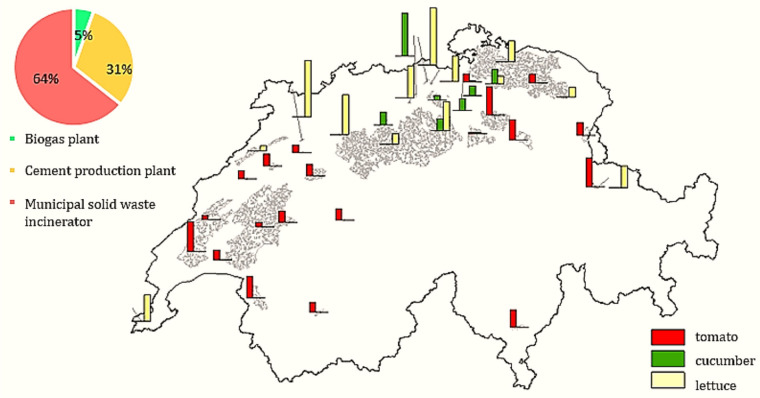
Opportunity map for replacing imported vegetables (tomato, cucumber, and lettuce) with domestic greenhouse crops to implement a local food strategy or zero import scenario (size of greenhouses [ha]) and waste heat source contribution. The underlying data can be found in Supporting Information S2, Appendix SI-6.

Figure 5 displays the opportunity map, whereby two factors have a key role: the energy demand of greenhouses in suitable land and the waste heat potential of heat suppliers. Consequently, the optimal points that are the output of our optimization framework are determined. Most selected sites belong to either the country's north or the west, as both suitable lands and waste heat resources are available. It should be noted that most of the model's greenhouse heat demand is satisfied by MSWIs and cement production plants (95%). MSWIs have the largest contribution since suitable lands are around their locations.

Since the peak heat demand for tomato and cucumber crops is higher than lettuce, the optimization model prioritizes tomato and cucumber over lettuce greenhouses and assigns suitable land that is close to waste heat suppliers to tomato and cucumber greenhouses. The study's results show that the annual total cost of implementing industrial symbiosis, including the expenses of establishing pipelines and transporting hot water, amounts to 532,080 CHF for 72 ha of tomato greenhouses, 221,760 CHF for 30 ha of cucumber greenhouses, and 694,617 CHF for lettuce greenhouses. When this and the additional investment cost for the heat recovery system (300,000 CHF per MW; Teke et al., 2010) are converted to per-kg costs, the overall total costs amount to 0.096 CHF/kg tomato, 0.063 CHF/kg cucumber, and 0.010 CHF/kg lettuce. To conduct a comparative analysis between the aforementioned estimation and the heat supply derived from a fossil fuel source, specifically natural gas, the annual greenhouse heat demand is calculated using reported data from European nations such as Germany and the Netherlands. The energy requirements for tomato production range between 12,600 and 14,990 GJ per hectare, while for cucumber cultivation, the range is between 13,000 and 14,245 GJ per hectare. The energy needed for lettuce growing is reported at 2820 GJ per hectare (Paris et al., 2022). Additionally, considering an investment cost of 60 CHF/kW for a boiler (Mohebi & Roshandel, 2023), the calculated yearly heating costs for tomato, cucumber, and lettuce greenhouses amount to 649,916, 632,914, and 131,762 CHF per hectare, respectively (or 1.08 CHF/kg tomato, 0.84 CHF/kg cucumber, and 0.32 CHF/kg lettuce). Therefore, waste-heat use is several orders of magnitude cheaper than fossil heating per kg of crop produced, making this option economically very attractive.

Ultimately, using a pipeline for direct heat transfer appears as the most advantageous alternative, primarily driven by the substantial investment expenses associated with ORC technology and the limited availability of waste heat resources. In the scenario where the electricity prices experience a three-fold increase, resulting in an estimated value of 0.5 CHF/kWh, the decision would be made in favor of the ORC system, which would provide a total power generating capacity of 5 MW. Additional information may be found in Supporting Information S1,Appendix SI-3.

### Sensitivity analysis

**FIGURE 6:**
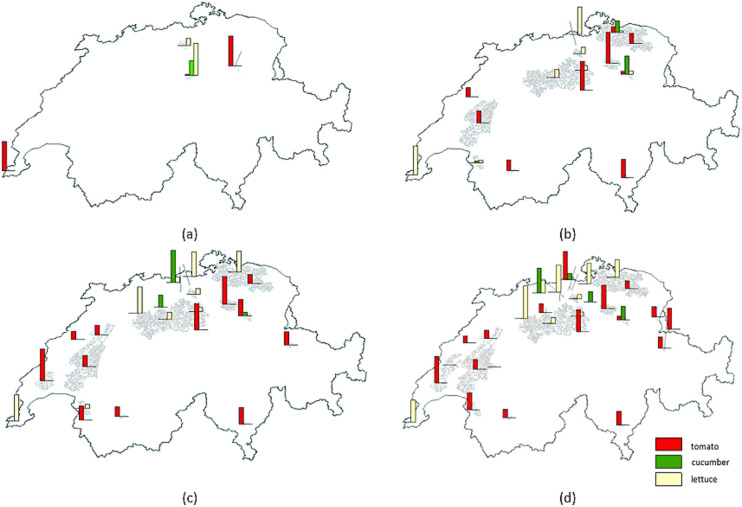
Locations of possible greenhouses and vegetables for (a) 20 ha, (b) 60 ha, (c) 100 ha, and (d) 140 ha. The underlying data can be found in Supporting Information S2, Appendix SI-7.

**FIGURE 7:**
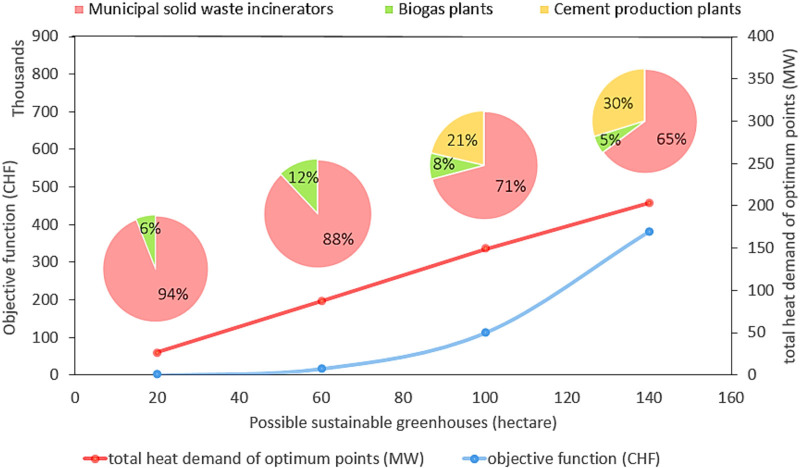
Effect of targeted greenhouse area including 20, 60, 100, and 140 ha on the objective function value and total utilized waste heat in optimum points and waste heat source contributions. The underlying data can be found in Supporting Information S2, Supporting Appendix SI-8.

In Section 3.3, a zero-import scenario was considered and ${\mathrm{T}}{{{\mathrm{A}}}_l}$ was set to satisfy this requirement. Here, the optimization model's response to changing vegetable demand is evaluated. Figure 6 shows how optimal points are distributed by increasing the targeted greenhouse area from 20 to 140 ha, and Figure 7 displays how the waste heat sources contribute to the completion of the aforementioned targeted greenhouses. Up to 60 ha, only MSWIs and biogas plants are chosen as optimum suppliers because of their geographic location and waste heat potentials. The cement production plants are added to meet the heat requirement when increasing the targeted greenhouse area. The total heat utilized in the optimum points rises quite linearly with the greenhouse area (as could be expected). In terms of costs, the low-cost options are exploited before the more expensive options, leading to an exponential-type curve for the objective function value (Figure 7). In other words, at first, the increment rate of the objective function is low, but from 60 ha, the slope dramatically increases. The reason for the linear behavior of the total heat utilized is that the optimization model found locations with fairly the same heat demand. Thus, the total heat utilized increased linearly (the north and west climates are similar in Switzerland, which similarly affects the peak heat demand of possible agricultural greenhouse that could be developed in those locations). The objective function of the initial 20 ha was zero, which means the optimization model can find waste heat sources that are exactly located on suitable lands (co-location advantage). Being zero objective function does not mean that this is for free, since here some costs, which are the same fixed for all cases, are neglected (such as greenhouse structure, heat recovery system, and local heat transfer).

## DISCUSSION AND CONCLUSION

The main objective of this study is to develop an effective decision-making tool tailored for national policymakers, facilitating governance of the food system to enhance the production of greenhouse vegetables. Our work demonstrated the feasibility of a nationwide optimization for an industrial symbiosis of waste heat sources and greenhouse crop production. This achievement can empower legislative authorities to establish a new roadmap for greenhouse development fostering particularly cost-optimal locations of symbiosis opportunities. It involves incorporating pertinent technological advancements into greenhouse practices, as well as formulating a comprehensive strategy to address challenges related to vegetable imports. For this aim, this study employed the industrial symbiosis concept to benefit the agricultural industry, specifically greenhouses, regarding resource conservation and suggested an optimization methodology. The opportunity maps were created to emphasize the locations that are most suitable for the establishment of waste heat-supplied greenhouses. This approach has the potential to assist policymakers in discerning the feasibility of adopting a local vegetable-sourcing strategy that utilizes waste heat sources, yielding positive outcomes for both the environment and the economy to further expand successful projects like Hinwil waste heat supplied greenhouse (Chertow, 2000). First, target industries and suitable greenhouse land areas were determined and mapped in a GIS for this aim. Then, analyses were carried out to determine the energy requirements of various crops grown in greenhouses on each suitable land and the waste heat potential of various industrial sources (MSWIs, cement production plants, and biogas plants). The maps of suitable land areas and waste heat availability were used as inputs to the economic optimization, which also took into account technical considerations and economy-of-scale relationships.

The main results of the study are:

The total waste heat potential from MSWIs, cement, and biogas plants that could be supplied to greenhouses amounts to 300 MW, where MSWIs account for 60% of total waste heat potential. The waste heat supplier map reveals to industry stakeholders (of MSWIs, cement, and biogas plants) the considerable potential of waste heat for effective utilization within networks of industrial symbiosis, specifically greenhouse heat demands in Switzerland. The results show that collaboration between selected industries and greenhouse operators to optimize waste heat utilization can lead to economic benefits for all stakeholders. This also confirms the necessity for innovative solutions that tap into existing waste heat resources to meet greenhouse heat demands in a cost-effective and environmentally sustainable way. Therefore, the study emphasizes that exploiting waste heat resources within industrial symbiosis networks would improve the energy sector's sustainability and resilience.

This waste heat potential would be enough to implement a local vegetable-sourcing strategy for tomatoes, lettuce, and cucumbers (zero imports). Optimal points are located in lands with lower heat demand near major waste heat sources. MSWIs and biogas plants are prioritized due to suited geography in lower demand, while cement production plants are also selected to satisfy related energy requirements by raising the crop demand. Up to 20 ha, the optimization model offers co-located optimal points without extra investment costs, mostly satisfied by MSWIs. However, by increasing the targeted greenhouse areas, the corresponding cost rises. The optimization findings can benefit food retailers and food supply chain managers by evaluating the economic impact of locally grown vegetables in waste heat-supplied greenhouses compared to other alternatives (e.g., imported vegetables). By understanding the trade-offs, they can make informed purchasing and marketing decisions. Furthermore, agricultural experts and greenhouse investors focused on environmentally friendly greenhouse crop production can utilize the greenhouse heat demand map to identify the most cost-effective locations for developing waste heat-supplied greenhouses.

Selected ORC capacities are not competitive cost-wise under current price conditions but would be selected as optimum technology if the prices more than triple. ORC would then only be implemented at some cement production plants with higher waste heat potential. In this case, electricity is also generated (5 MW) in addition to satisfying energy demand. This emphasizes the decisive influence of the electricity price on the adoption of ORC technology for agricultural greenhouses that utilize waste heat. Additionally, optimization results regarding ORC shed light on how this technology might be effectively integrated into local greenhouse systems for both energy service companies and electricity retailers. ORC-generated electricity can offset some of the electricity demands of agricultural greenhouse systems (e.g., supplementary lighting). Note that the scale of ORC can influence the total cost of this technology, with costs decreasing with increasing plant size.

Furthermore, the technical details of the industrial symbiosis network between waste heat suppliers and greenhouse systems (heat exchanger design, pipeline isolation material, and heat distribution system in the greenhouse) can enhance overall efficiency levels in capturing, transportation, and distribution. However, this enhancement typically induces higher total costs.

While our optimization model is designed to apply to a broad range of industrial symbiosis scenarios, it is essential to recognize that the impact of regional regulations may require more focused investigation in specific cases. However, the purpose of our study is to demonstrate the potential for policy-making as a whole, rather than focusing on particular cantonal legislation.

Finally, it is worth mentioning that the optimization framework was developed in a way that can fit according to the considered application. In this study, Switzerland was selected as a developed country to show how industrial symbiosis can help supply waste heat to greenhouses. However, any region with diverse contexts can be a case study for this purpose.

To implement the study findings, several strategies could be considered. This paper demonstrates that in many locations, the production of vegetables in greenhouses that utilize locally sourced waste heat is cost-effective. It should be noted that the scale of the greenhouse system influences the overall cost of the greenhouse project. As the size of the greenhouse increases, the total cost of the symbiosis network (e.g., pipeline investment cost) tends to decrease due to the economy of scale.

These findings should be disseminated to investors, industries that provide waste heat, and farmers. For example, workshops to disseminate the accomplishments can unveil potential collaborations between waste heat suppliers and operators of agricultural greenhouse systems. These workshops can serve as a platform for knowledge exchange and networking, facilitating the identification of synergies and partnerships. Through collaboration and knowledge sharing, participants (e.g., investors, waste heat suppliers, and farmers) can explore new opportunities for developing waste heat-supplied greenhouses. Furthermore, the tools developed in this study, specifically the greenhouse heat demand model and optimization framework, can be shared with interested stakeholders and be refined or applied to other regions.

The Swiss energy strategy for protected cultivation, which indicates the transition to non-fossil fuel greenhouses by the year 2040 (Der Verband Schweizer Gemüseproduzenten (VSGP), n.d.), facilitates and supports the implementation of waste heat supplied-greenhouses proposed in this study. However, modifying rules or creating incentives provided by authorities to remove barriers (Czyżewski et al., 2021; Energie-investeringsaftrek (EIA) voor Ondernemers. RVO.nl, n.d.; Pretty et al., 2002) and encourage the expansion of waste-heat driven agricultural greenhouses would help foster industrial symbiotic relationships. To encourage greenhouse investors to utilize waste heat-supplied greenhouse systems, it is essential to make adjustments to land use regulations and permissions regarding greenhouse construction. Concerns from local communities regarding landscape changes, soil protection, or other environmental impacts can hinder project acceptance and implementation. Therefore, careful planning, transparent community engagement, and adaptable supportive regulations are crucial for successfully executing such projects. All in all, the involvement of the public, stakeholders, and authorities is essential in attaining sustainable goals for local production (Reddy, 2016). Finally, consumers need to be informed and potential acceptance issues addressed (Vlaeminck et al., 2014) (e.g., potential objections toward food produced in the vicinity of a waste incineration plant). Many supermarkets in Switzerland already use labels to mark products with high or low environmental impacts. Therefore, a life-cycle assessment study could be performed on the crops produced with waste heat and the findings could be integrated into the existing labeling systems, fostering the consumption of sustainable products. Also, one of the barriers to conducting industrial symbiosis is lacking trust and how the reciprocal relationship should be managed for a long period (such as how greenhouse owners might face industry shutdowns) (Golev et al., 2015). To address this concern, one possible solution is to establish an “insurance” system that helps mitigate risks and build confidence among participants.

## LIMITATIONS AND FUTURE WORK

Although this paper is more comprehensive than previous work published, it still worked with many simplifications and has several limitations that should be palliated in future research. This includes assumptions of model linearity and the neglect of land costs and a potential future carbon tax. Moreover, we only assessed one very conservative scenario of meeting peak heat demand, which results in unused waste heat surplus. Alternatively, scenarios that use waste heat in combination with primary heat sources could be constructed. This includes maximizing waste heat utilization, considering the seasonality of supply and demand, and covering some supply deficits with primary heat sources (e.g., biomass). Additionally, surplus waste heat could be used to increase indoor temperatures beyond the set point (to increase yields), and indoor temperatures could be lowered in times of waste-heat shortage. To tackle this problem, future research needs to focus on developing a dynamic model and incorporating thermal storage to enhance waste heat consumption. Furthermore, current greenhouses in Switzerland are only operated for part of the year (sparing the coldest months), while in this paper year-round production was assumed. It should be also noted that replacing all imports with local production is a theoretical modeling scenario in this study (obviously, this assumption could easily be changed with any scenario, e.g., half of the demand).

Landforms including artificial surfaces, forests and semi-natural areas, wetlands, and water bodies unsuitable for agricultural greenhouses were excluded from the spatial analysis performed for this study. Additionally, legal constraints need to be systematically considered in subsequent assessments, including national as well as cantonal (sub-national) restrictions for greenhouse construction.

This study specifically focuses on new greenhouses. However, it is noteworthy that existing greenhouses currently powered by fossil fuels may also consider transitioning to utilize waste heat. A detailed analysis of this competition is earmarked as future work.

Providing supplementary lighting in greenhouses may pose a challenge regarding electricity consumption, potentially impacting the seasonal performance and overall quality of vegetable output. To address this, the integration of supplementary lighting needs, dependent on natural solar irradiation on eligible lands, along with the heat demand, will be incorporated into the optimization model in future research.

## Supplementary Information

**Supporting Information S1**: The Supporting Information S1 describes how the optimization model is formulated when the concept of economy of scale is taken into account (Appendix SI-1). In addition, the complete list of the coefficients of the optimization model and their values for selected waste heat sources are provided (Appendix SI-2). Furthermore, the SI gives additional results of suitable land analysis, economy of scale, waste heat suppliers and the ORC system (Appendix SI-3).

**Supporting Information S2**: Also, Supporting Information S2 provides the locations and practical waste heat potential of selected suppliers in Switzerland (Appendix SI-4); peak heat demand for suitable lands for tomato, cucumber, and lettuce greenhouses in Switzerland (Appendix SI-5); opportunity map for replacing imported vegetables (tomato, cucumber, and lettuce) with domestic greenhouse crops (Appendix SI-6); locations of possible greenhouses and vegetables for 20 hectares, 60 hectares, 100 hectares and 140 hectares scenarios (Appendix SI-7); effect of targeted greenhouse area including 20, 60, 100, and 140 ha on the objective function value and total utilized waste heat in optimum points and waste heat source contributions (Appendix SI-8).

## Data Availability

The data that support the findings of this study are available in the supporting information of this article.
