# Supplementary material for: Spatial optimization of industrial symbiosis for heat supply of agricultural greenhouses
Source: J Ind Ecol. 2024 Aug 13;28(6):1507–23. doi: 10.1111/jiec.13543 (PMC11667671; doi:10.1111/jiec.13543)
Supplement: Supplementary file 1 — Supporting Information S1: The Supporting Information S1 describes how the optimization model is formulated when the concept of economy of scale is taken into account (Appendix SI-1). In addition, the complete list of the coefficients of the optimization model and their values for selected waste heat sources are provided (Appendix SI-2). Furthermore, the SI gives additional results of suitable land analysis, economy of scale, waste heat suppliers and the ORC system (Appendix SI-3). [file 44498_2024_2806014_MOESM1_ESM.docx]

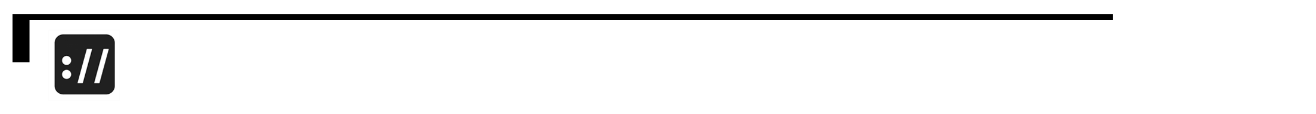


SUPPORTING INFORMATION FOR:

Rezaei, F., Burg, V. , Pfister, S. , Hellweg, S. & Roshandel, R. (2024.) Spatial Optimization of Industrial Symbiosis for Heat Supply of Agricultural Greenhouses. *Journal of Industrial Ecology.*


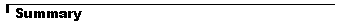


The supporting information 1 describes how the optimization model is formulated when the concept of economy of scale is taken into account (Appendix SI-1); the complete list of the coefficient of optimization model and their values for selected waste heat sources (Appendix SI-2); additional results of suitable land analysis, economy of scale, waste heat suppliers and the ORC system (Appendix SI-3).


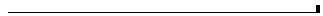


Table of Contents

[Appendix SI-1 Economy of scale in optimization formulation 2](#_Toc164861340)

[Appendix SI-2 Optimization model coefficients and their values for selected sources 7](#_Toc164861341)

[Appendix SI-3 Additional results 9](#_Toc164861342)

[References 13](#_Toc164861343)

### Appendix SI-1 Economy of scale in optimization formulation

To evaluate how economy of scale affects the optimization formulation, first the basic formulation is presented. Then this formulation is integrated with economy of scale considerations.

- 1. **Basic formulation**

For basic formulation, the objective function is as Eq. 1. Term 1 is the investment cost of piping for transferring heat where$INV{, d}_{i,j}, Y_{i,j}$ and $CRF$ denote capital cost of piping ($\frac{CHF}{km}$), distance between source $i$ and demand $j$ $(km)$, binary variable to show which pathway is selected and capital recovery factor (to calculate the present value of an annuity), respectively. Term 2 represents the operational cost of pump power for transferring heat where $C_{OP}, {PD}_{l}$ and ${CF}_{1}$ denote operation cost coefficient ($\frac{CHF}{km.MWh}$), peak thermal energy needed for greenhouse area $(MW/ha)$ and the number of hours in one year that greenhouse needs heat $(h)$, respectively. Term 3 denotes the investment cost of waste heat to electricity technology (ORC) where ${SC}_{ORC}$ and ${CAP_{orc}}_{i,j}$ are the investment cost of ORC technology ($\frac{CHF}{MW}$) and ORC capacity $(MW)$. Term 4 is the maintenance cost of waste heat to electricity technology where ${OM}_{ORC}$ and ${CF}_{2}$ is maintenance coefficient ($\frac{CHF}{MWh}$) and capacity factor of ORC technology (h/year), respectively. Finally, the last term is the income from the sale of electricity generated from waste heat to electricity technology (ORC) to the grid where ${Pr}_{el}$ is the price of selling electricity ($\frac{CHF}{MWh}$).

| $Min total annual Cost=\sum_{i=1}^{I} \sum_{j=1}^{J} {INV\times d}_{i,j}\times Y_{i,j}\times CRF+\sum_{k=1}^{K} \sum_{i=1}^{I} \sum_{j=1}^{J} \sum_{l=1}^{L} {C_{OP}\times d}_{i,j}\times X_{i,j,l,k}\times{PD}_{l}\times{CF}_{1}+\sum_{i=1}^{I} \sum_{j=1}^{J} {SC}_{ORC}\times{CAP_{orc}}_{i,j} \times CRF+ \sum_{i=1}^{I} \sum_{j=1}^{J} {OM}_{ORC}\times{CAP_{orc}}_{i,j} \times{CF}_{2}- \sum_{i=1}^{I} \sum_{j=1}^{J} {{Pr}_{el}\times CAP_{orc}}_{i,j}\times{CF}_{2}$ | (Eq. 1) |
| --- | --- |

Set of constraints regarded for the basic formulation are as Eqs. 2-6. Eq. 2 is defined to ensure that related greenhouse areas (${TA}_{l}$) are satisfies for all vegetables including tomato, cucumber and lettuce. Eq. 3 guarantees that the total area of greenhouses is lower than the suitable land area in a specific location where $A_{j}$ is the available suited agricultural area in the region. Eq. 4 requires that the peak heat demand must be less than the total waste heat potential of industries where $hlc$ and $E_{i}$ denote heat loss coefficient coming from heat transferring$(\frac{\%}{km}$) and waste heat potential $(MW)$. Eq. 5 identifies which pathway is selected as optimum (by assigning 1 to $Y_{i,j}$) for investment cost of heat transferring. If $X_{i,j,l,k}$ has value greater than zero, the corresponding $Y_{i,j}$ should be 1 otherwise it is zero and Big M is a large value for making it feasible. Eq. 6 is also defined to restrict ORC capacity due to available waste heat on the condition that ORC is chosen by the model, where the ${ratio}_{HE}$ denotes the ratio of heat to electricity in ORC system .

| $\sum_{\boldsymbol{i}=\boldsymbol{1}}^{\boldsymbol{I}} \sum_{\boldsymbol{j}=\boldsymbol{1}}^{\boldsymbol{J}} \sum_{\boldsymbol{k}=\boldsymbol{1}}^{\boldsymbol{K}} \boldsymbol{X}_{\boldsymbol{i},\boldsymbol{j},\boldsymbol{l},\boldsymbol{k}}\geq\boldsymbol{TA}_{\boldsymbol{l}}$ | (Eq. 2) |
| --- | --- |
| $\sum_{\boldsymbol{i}=\boldsymbol{1}}^{\boldsymbol{I}} \sum_{\boldsymbol{l}=\boldsymbol{1}}^{\boldsymbol{L}} \sum_{\boldsymbol{k}=\boldsymbol{1}}^{\boldsymbol{K}} \boldsymbol{X}_{\boldsymbol{i},\boldsymbol{j},\boldsymbol{l},\boldsymbol{k}}\leq\boldsymbol{A}_{\boldsymbol{j}}$ | (Eq. 3) |
| $\sum_{\boldsymbol{j}=\boldsymbol{1}}^{\boldsymbol{J}} \sum_{\boldsymbol{l}=\boldsymbol{1}}^{\boldsymbol{L}} \sum_{\boldsymbol{k}=\boldsymbol{1}}^{\boldsymbol{K}} \boldsymbol{X}_{\boldsymbol{i},\boldsymbol{j},\boldsymbol{l},\boldsymbol{k}}\times\boldsymbol{PD}_{\boldsymbol{l},\boldsymbol{j}}\times(\boldsymbol{1}+\boldsymbol{hlc}\times\boldsymbol{d}_{\boldsymbol{i},\boldsymbol{j}})\leq\boldsymbol{E}_{\boldsymbol{i}}$ | (Eq. 4) |
| $\sum_{\boldsymbol{k}=\boldsymbol{1}}^{\boldsymbol{K}} \sum_{\boldsymbol{l}=\boldsymbol{1}}^{\boldsymbol{L}} \boldsymbol{X}_{\boldsymbol{i},\boldsymbol{j},\boldsymbol{l},\boldsymbol{k}}-\boldsymbol{Y}_{\boldsymbol{i},\boldsymbol{j}}\times\boldsymbol{Big} \boldsymbol{M}\leq\boldsymbol{0}$ | (Eq. 5) |
| ${\boldsymbol{CA}\boldsymbol{P}_{\boldsymbol{orc}}}_{\boldsymbol{i},\boldsymbol{j}}^{\boldsymbol{m}}\leq\frac{\sum_{\boldsymbol{l}=\boldsymbol{1}}^{\boldsymbol{L}} \left( \boldsymbol{X}_{\boldsymbol{i},\boldsymbol{j},\boldsymbol{l},\boldsymbol{2}}\times\boldsymbol{PD}_{\boldsymbol{l},\boldsymbol{j}} \right)}{\boldsymbol{ratio}_{\boldsymbol{HE}}}$ | (Eq. 6) |

- 1. **Economy of scale**

As shown in Fig. 1, the investment cost of piping does not increase linearly, so that the larger the pipeline capacity, the lower the unit cost of output. To respond to this problem, regarding particular pipe diameters and corresponding energy carried by them, the investment cost of piping is discretized into specific ranges of heat capacity.


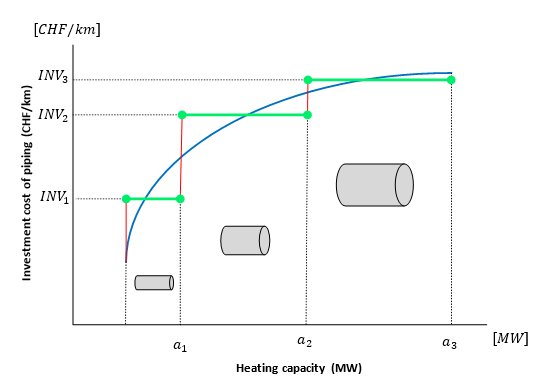


Fig. 1. Discretization of investment cost of piping according to heat capacity assigned to specific pipe

The information for the curve was extracted from real data (for this case, the report by SFOE [62]). Then, this curve has been broken into three intervals (sections). For each section, the specific pipe with a specific diameter has been considered, and in this way, the curve has been converted into a step diagram used for linearizing. To adjust the investment cost of piping, the objective function needs to be extended with a new term multiplying discretized investment cost of piping ($\sum_{n=1}^{N} {INV}_{n}$) and binary variables ($t_{i,j}^{n}$) for selecting appropriate technical characteristics based on the heat capacity interval (Eq. 7). N denotes the number of intervals of the pipeline heat capacity. In terms of constraints Eqs. 8 and 9 are added to the basic formulation. Eq. 8 identifies the heating capacity ($\sum_{n=1}^{N} a_{n}t_{i,j}^{n}$) for the selected pathway according to range of heating carried by the pipeline for optimum greenhouse, and Eq. 9 means only one pathway can be selected$.$

| $discretized piping investment cost term in objective function =\sum_{i=1}^{I} \sum_{j=1}^{J} \sum_{n=1}^{N} {INV}_{n}{\times t_{i,j}^{n}\times d}_{i,j}\times CRF$ | (Eq. 7) |  |
| --- | --- | --- |
| $\sum_{l=1}^{L} X_{i,j,l,1}\times{PD}_{l,j}\leq\sum_{n=1}^{N} a_{n}t_{i,j}^{n} n=1,\ldots, N$ | (Eq. 8) |  |
| $\sum_{n=1}^{N} t_{i,j}^{n}=1 t_{i,j}^{n}\epsilon\{0,1\}$ | (Eq. 9) | |

The economy of scale also considerably affects the investment and maintenance cost of ORC. As the system capacity rises, investment and maintenance unit costs of ORC technology decline, as demonstrated in Fig. 2.


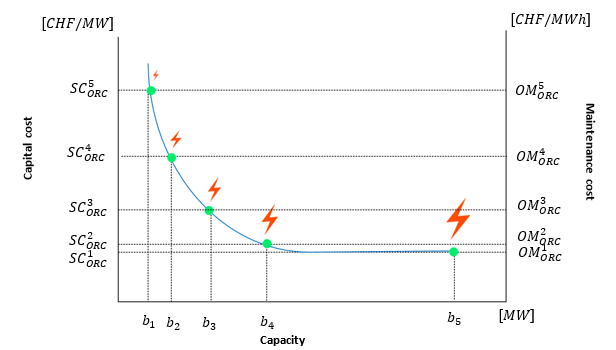


Fig. 2. Discretization of capital and maintenance cost of ORC according to ORC capacity range

As illustrated in Fig. 2, the same strategy applied for linearizing investment cost of piping is used here again. In this case, new investment and operation costs of ORC (Eq. 10 and 11) and the profit of selling electricity to the grid (Eq. 12) are used instead of related terms in the basic formulation of the objective function. ${CAP_{orc}}_{i,j}^{m}$ is ORC capacity according to discretized ranges. M is the number of discrete ranges of ORC capacity. All parameter values for selected case study are listed in section 2.2.2.

| $discretized investment cost of ORC=\sum_{i=1}^{I} \sum_{j=1}^{J} \sum_{m=1}^{M} {SC}_{ORC}^{m}{\times CAP_{orc}}_{i,j}^{m}\times CRF$ | (Eq. 10) |
| --- | --- |
| $discretized maintenance cost of ORC=\sum_{i=1}^{I} \sum_{j=1}^{J} \sum_{m=1}^{M} {OM}_{ORC}^{m}{\times CAP_{orc}}_{i,j}^{m}\times{CF}_{2}$ | (Eq. 11) |
| $discretized income of ORC=\sum_{m=1}^{M} \sum_{i=1}^{I} \sum_{j=1}^{J} {{Pr}_{el}\times CAP_{orc}}_{i,j}^{m}\times{CF}_{2}$ | (Eq. 12) |

Considering constraints, Eq. 6 is replaced by Eqs. 13-17. Eq. 13 identifies which range of ORC capacity ($\sum_{m=1}^{M} b_{m}z_{i,j}^{m}$) is selected where $z$ is a binary variable for choosing one pathway. Eq. 14 valorizes the related binary variable for the optimum pathway. Eq. 16 and 17 assign the suited ORC capacity according to heat demand needed for the greenhouse.

| $\frac{\sum_{\boldsymbol{l}=\boldsymbol{1}}^{\boldsymbol{L}} \boldsymbol{X}_{\boldsymbol{i},\boldsymbol{j},\boldsymbol{l},\boldsymbol{2}}\times\boldsymbol{PD}_{\boldsymbol{l},\boldsymbol{j}}}{\boldsymbol{ratio}_{\boldsymbol{HE}}}=\sum_{\boldsymbol{m}=\boldsymbol{1}}^{\boldsymbol{M}} \boldsymbol{b}_{\boldsymbol{m}}\boldsymbol{z}_{\boldsymbol{i},\boldsymbol{j}}^{\boldsymbol{m}} \boldsymbol{m}=\boldsymbol{1},\ldots, \boldsymbol{M}$ | (Eq. 13) |
| --- | --- |
| $\sum_{\boldsymbol{m}=\boldsymbol{1}}^{\boldsymbol{M}} \boldsymbol{z}_{\boldsymbol{i},\boldsymbol{j}}^{\boldsymbol{m}}=\boldsymbol{1} \boldsymbol{z}_{\boldsymbol{i},\boldsymbol{j}}^{\boldsymbol{m}}\boldsymbol{\epsilon} \{\boldsymbol{0},\boldsymbol{1}\}$ | (Eq. 14) |
| ${\boldsymbol{CA}\boldsymbol{P}_{\boldsymbol{orc}}}_{\boldsymbol{i},\boldsymbol{j}}^{\boldsymbol{m}}\leq\frac{\sum_{\boldsymbol{l}=\boldsymbol{1}}^{\boldsymbol{L}} \left( \boldsymbol{X}_{\boldsymbol{i},\boldsymbol{j},\boldsymbol{l},\boldsymbol{2}}\times\boldsymbol{PD}_{\boldsymbol{l},\boldsymbol{j}} \right)}{\boldsymbol{ratio}_{\boldsymbol{HE}}}$ | (Eq. 16) |
| ${\boldsymbol{CA}\boldsymbol{P}_{\boldsymbol{orc}}}_{\boldsymbol{i},\boldsymbol{j}}^{\boldsymbol{m}}\geq\frac{\sum_{\boldsymbol{l}=\boldsymbol{1}}^{\boldsymbol{L}} \left( \boldsymbol{X}_{\boldsymbol{i},\boldsymbol{j},\boldsymbol{l},\boldsymbol{2}}\times\boldsymbol{PD}_{\boldsymbol{l},\boldsymbol{j}} \right)}{\boldsymbol{ratio}_{\boldsymbol{HE}}}-(\boldsymbol{1}-\boldsymbol{z}_{\boldsymbol{i},\boldsymbol{j}}^{\boldsymbol{m}})\times\boldsymbol{Big} \boldsymbol{M}$ | (Eq. 17) |

In summary, the new objective function with considering economy of scale (Eqs. 17-7) is as Eq. 18 **(new terms are bold)**:

| $Min total annual Cost=\sum_{i=1}^{I} \sum_{j=1}^{J} \sum_{\boldsymbol{n=1}}^{\boldsymbol{N}} \boldsymbol{INV}_{\boldsymbol{n}}{\boldsymbol{\times}\boldsymbol{t}_{\boldsymbol{i,j}}^{\boldsymbol{N}}\times d}_{i,j}\times CRF+\sum_{k=1}^{K} \sum_{i=1}^{I} \sum_{j=1}^{J} \sum_{l=1}^{L} {C_{OP}\times d}_{i,j}\times X_{i,j,l,k}\times{PD}_{l}\times{CF}_{1}+\sum_{i=1}^{I} \sum_{j=1}^{J} \sum_{\boldsymbol{m=1}}^{\boldsymbol{M}} \boldsymbol{SC}_{\boldsymbol{ORC}}^{\boldsymbol{m}}{\boldsymbol{\times CA}\boldsymbol{P}_{\boldsymbol{orc}}}_{\boldsymbol{i,j}}^{\boldsymbol{m}}+ \sum_{i=1}^{I} \sum_{j=1}^{J} \sum_{\boldsymbol{m=1}}^{\boldsymbol{M}} \boldsymbol{OM}_{\boldsymbol{ORC}}^{\boldsymbol{m}}{\boldsymbol{\times CA}\boldsymbol{P}_{\boldsymbol{orc}}}_{\boldsymbol{i,j}}^{\boldsymbol{m}}\boldsymbol{\times}{CF}_{2}\boldsymbol{-} \sum_{i=1}^{I} \sum_{j=1}^{J} \sum_{\boldsymbol{m=1}}^{\boldsymbol{M}} {{Pr}_{el}\boldsymbol{\times CA}\boldsymbol{P}_{\boldsymbol{orc}}}_{\boldsymbol{i,j}}^{\boldsymbol{m}}\boldsymbol{\times}{CF}_{2}$  $\boldsymbol{n=1,\ldots,N \& m=1,\ldots, M}$ | (Eq. 18) |
| --- | --- |

All constraints for this purpose are put forward as below (Eqs. 19-27):

| $\sum_{\boldsymbol{i}=\boldsymbol{1}}^{\boldsymbol{I}} \sum_{\boldsymbol{j}=\boldsymbol{1}}^{\boldsymbol{J}} \sum_{\boldsymbol{k}=\boldsymbol{1}}^{\boldsymbol{K}} \boldsymbol{X}_{\boldsymbol{i},\boldsymbol{j},\boldsymbol{l},\boldsymbol{k}}\geq\boldsymbol{TA}_{\boldsymbol{l}}$ | (Eq. 19) | | |
| --- | --- | --- | --- |
| $\sum_{\boldsymbol{i}=\boldsymbol{1}}^{\boldsymbol{I}} \sum_{\boldsymbol{l}=\boldsymbol{1}}^{\boldsymbol{L}} \sum_{\boldsymbol{k}=\boldsymbol{1}}^{\boldsymbol{K}} \boldsymbol{X}_{\boldsymbol{i},\boldsymbol{j},\boldsymbol{l},\boldsymbol{k}}\leq\boldsymbol{A}_{\boldsymbol{j}}$ | (Eq. 20) | | |
| $\sum_{\boldsymbol{j}=\boldsymbol{1}}^{\boldsymbol{J}} \sum_{\boldsymbol{l}=\boldsymbol{1}}^{\boldsymbol{L}} \sum_{\boldsymbol{k}=\boldsymbol{1}}^{\boldsymbol{K}} \boldsymbol{X}_{\boldsymbol{i},\boldsymbol{j},\boldsymbol{l},\boldsymbol{k}}\times\boldsymbol{PD}_{\boldsymbol{l},\boldsymbol{j}}\times(\boldsymbol{1}+\boldsymbol{hlc}\times\boldsymbol{d}_{\boldsymbol{i},\boldsymbol{j}})\leq\boldsymbol{E}_{\boldsymbol{i}}$ | (Eq. 21) | | |
| $\sum_{\boldsymbol{l}=\boldsymbol{1}}^{\boldsymbol{L}} \boldsymbol{X}_{\boldsymbol{i},\boldsymbol{j},\boldsymbol{l},\boldsymbol{1}}\times\boldsymbol{PD}_{\boldsymbol{l},\boldsymbol{j}}\leq\sum_{\boldsymbol{n}=\boldsymbol{1}}^{\boldsymbol{N}} \boldsymbol{a}_{\boldsymbol{n}}\boldsymbol{t}_{\boldsymbol{i},\boldsymbol{j}}^{\boldsymbol{n}} \boldsymbol{n}=\boldsymbol{1},\ldots, \boldsymbol{N}$ | | (Eq. 22) |  |
| $\sum_{\boldsymbol{n}=\boldsymbol{1}}^{\boldsymbol{N}} \boldsymbol{t}_{\boldsymbol{i},\boldsymbol{j}}^{\boldsymbol{n}}=\boldsymbol{1} \boldsymbol{t}_{\boldsymbol{i},\boldsymbol{j}}^{\boldsymbol{n}}\boldsymbol{\epsilon} \{\boldsymbol{0},\boldsymbol{1}\}$ | | (Eq. 23) |  |
| $\frac{\sum_{\boldsymbol{l}=\boldsymbol{1}}^{\boldsymbol{L}} \boldsymbol{X}_{\boldsymbol{i},\boldsymbol{j},\boldsymbol{l},\boldsymbol{2}}\times\boldsymbol{PD}_{\boldsymbol{l},\boldsymbol{j}}}{\boldsymbol{ratio}_{\boldsymbol{HE}}}\leq\sum_{\boldsymbol{m}=\boldsymbol{1}}^{\boldsymbol{M}} \boldsymbol{b}_{\boldsymbol{m}}\boldsymbol{z}_{\boldsymbol{i},\boldsymbol{j}}^{\boldsymbol{m}} \boldsymbol{m}=\boldsymbol{1},\ldots, \boldsymbol{M}$ | | (Eq. 24) |  |
| $\sum_{\boldsymbol{m}=\boldsymbol{1}}^{\boldsymbol{M}} \boldsymbol{z}_{\boldsymbol{i},\boldsymbol{j}}^{\boldsymbol{m}}=\boldsymbol{1} \boldsymbol{z}_{\boldsymbol{i},\boldsymbol{j}}^{\boldsymbol{m}}\boldsymbol{\epsilon} \{\boldsymbol{0},\boldsymbol{1}\}$ | | (Eq. 25) |  |
| ${\boldsymbol{CA}\boldsymbol{P}_{\boldsymbol{orc}}}_{\boldsymbol{i},\boldsymbol{j}}^{\boldsymbol{m}}\leq\frac{\sum_{\boldsymbol{l}=\boldsymbol{1}}^{\boldsymbol{L}} \left( \boldsymbol{X}_{\boldsymbol{i},\boldsymbol{j},\boldsymbol{l},\boldsymbol{2}}\times\boldsymbol{PD}_{\boldsymbol{l},\boldsymbol{j}} \right)}{\boldsymbol{ratio}_{\boldsymbol{HE}}}$ | | (Eq. 26) |  |
| ${\boldsymbol{CA}\boldsymbol{P}_{\boldsymbol{orc}}}_{\boldsymbol{i},\boldsymbol{j}}^{\boldsymbol{m}}\geq\frac{\sum_{\boldsymbol{l}=\boldsymbol{1}}^{\boldsymbol{L}} \left( \boldsymbol{X}_{\boldsymbol{i},\boldsymbol{j},\boldsymbol{l},\boldsymbol{2}}\times\boldsymbol{PD}_{\boldsymbol{l},\boldsymbol{j}} \right)}{\boldsymbol{ratio}_{\boldsymbol{HE}}}-(\boldsymbol{1}-\boldsymbol{z}_{\boldsymbol{i},\boldsymbol{j}}^{\boldsymbol{m}})\times\boldsymbol{Big} \boldsymbol{M}$ | | (Eq. 27) |  |

### Appendix SI-2 Optimization model coefficients and their values for selected sources

Table 1 displays the coefficients for practical waste heat potential estimation for selected sources.

| Table 1. coefficients for practical waste heat potential estimation for selected sources | | | | | | |
| --- | --- | --- | --- | --- | --- | --- |
| **Sector** | **Parameter** | **Symbol** | **Value** | **Unit** | **Ref.** |  |
| **Cement production plants** | specific energy consumption | $SEC$ | 3.53 | $\frac{GJ}{tonne clinker}$ | [1] |  |
|  | stack loss | $\alpha$ | 23 | % | [2] |  |
|  | cooler loss | $\beta$ | 11 | % |  |  |
|  | Practical heat recovery factor | $\eta_{c}$ | 50 | % | Assumed*** |  |
|  | clinker production | ${Ca}_{c}$ | 5,000,000* | tonne | [1] |  |
| **Municipal solid waste incinerators** | solid waste entering to boiler | $SW$ | 4,071,643* | tonne | [3] |  |
|  | utilized as district heating | $CH$ | 3,929,310* | MWh |  |  |
|  | self-consumption of heat | $SC$ | 337,020* | MWh |  |  |
|  | electricity sold to grid | $EL$ | 1,855,486* | MWh |  |  |
|  | self-consumption of electricity | $SE$ | 492,298* | MWh |  |  |
|  | boiler efficiency | $e_{b}$ | 0.81** | % |  |  |
|  | Practical heat recovery factor | $\eta_{i}$ | 50 | % | Assumed*** |  |
| **Biogas plants** | ratio of heat recovery to electricity | $\tau_{H/E}$ | 1.5 | - | [4] |  |
|  | generated electricity | $EB$ | 47,340* | kW | [5] |  |
|  | minimum percentage of heat not used for the fermenter | $\varphi$ | 40 | % | [6] |  |
| * this value is the sum of the kiln capacity production in Switzerland, the individual plant capacity is used in our calculation  ** this is the average value for MSWI in Switzerland, but it might vary depending on the used technology and the age of the plants  *** this assumption corresponds to the best practice in Switzerland according to datasheets of heat recovery manufactures and the visit of case study sites | | | | | | |

Technical and economic parameters used in the optimization are displayed in Table 2.

| Table 2. Parameters utilized in the optimization formulation | | | | | |
| --- | --- | --- | --- | --- | --- |
| Parameter |  | Symbol | Unit | Value | Ref |
| Investment cost of piping for heat transferring and ORC | Capital cost | $L_{1}$  $L_{2}$  $L_{3}$ | $\frac{CHF}{m}$ | $462$  $974$  $1500$ | [7] |
|  | Power range | $a_{1}$  $a_{2}$  $a_{3}$ | $MW$ | $1.56$  $7.60$  $25.24$ |  |
| Operational cost of pump power for transferring heat | | $C_{\mathrm{op}}$ | $\frac{CHF}{ton.km}$ | $0.017$ | [8] |
| Selling price of electricity generated from ORC | | ${Pr}_{\mathrm{el}\text{ }}$ | $\frac{CHF}{GJ}$ | $42$ | [9] |
| Cost of ORC system | Capital cost | ${SC}_{ORC}^{1}$  ${SC}_{ORC}^{2}$  ${SC}_{ORC}^{3}$  ${SC}_{ORC}^{4}$  ${SC}_{ORC}^{5}$ | $\frac{CHF}{kW}$ | $11,000$  $9800$  $7350$  $6130$  $5150$ | [9] |
|  | Maintenance cost | ${OM}_{ORC}^{1}$  ${OM}_{ORC}^{2}$  ${OM}_{ORC}^{3}$  ${OM}_{ORC}^{4}$  ${OM}_{ORC}^{5}$ | $\frac{CHF}{GJ}$ | $13.89$  $10.28$  $8.89$  $8.33$  $6.94$ |  |
|  | Capacity | $b_{1}$  $b_{2}$  $b_{3}$  $b_{4}$  $b_{5}$ | $MW$ | $0.5$  $1$  $5$  $20$  $50$ |  |
| Discount rate for Switzerland | | $r$ | $\%$ | $10.5$ | [9] |
| Ratio of heat to electricity in ORC system | | ${ratio\text{ }}_{H/E}$ | $\frac{MWh}{MWh}$ | $4$ | [10-12] |
| Heat loss coefficient | | hlc | %/km | 5 | [8] |

### Appendix SI-3 Additional results

- 1. **. Suitable land analysis**

It was assumed that greenhouses can be constructed on agricultural land. The suitable land analysis was performed by using the Copernicus database to extract areas located within the agricultural category [13]. The land attributes including area of agricultural lands and the distances between waste heat sources and these lands are the inputs of the optimization problem. It should be mentioned that the land regulations in specific Cantons in Switzerland restricts greenhouse constructions on some areas. To tackle this challenge, our proposed framework is able to eliminate restricted lands using additional constraints in future research.

Our suitable land analysis identified 6414 contiguous land pieces in the range of 0.1 - 106 hectares. The size distribution of suitable contiguous land areas is depicted in Fig. 3. It can be seen that there are numerous suitable land areas that could be suited for developing large-scale greenhouses (2059 land plots with an area greater than 100 hectares). This can be advantageous when the economy of scale is considered. However, large-scale lands are usually owned by many owners (and not just one), which makes implementation of this consideration more difficult.


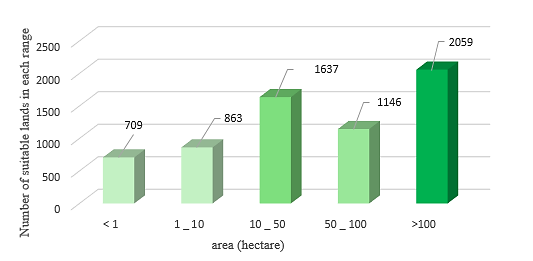


Fig. 3. Size distribution of suitable contiguous agricultural lands for developing greenhouses in Switzerland

Fig. 4 shows the total area in each Canton that is classified as suitable for developing greenhouses in Switzerland. The vast potential is located in the west and southwest parts of the country. For example, although Zurich and Ticino has the same area, Zurich has much more suitable land for developing greenhouses. More than 50% of total area of suitable lands are situated in only three cantons: Zurich, Bern, and Graubünden. These cantons additionally include many industrial waste heat suppliers which can cover energy demand of greenhouses.


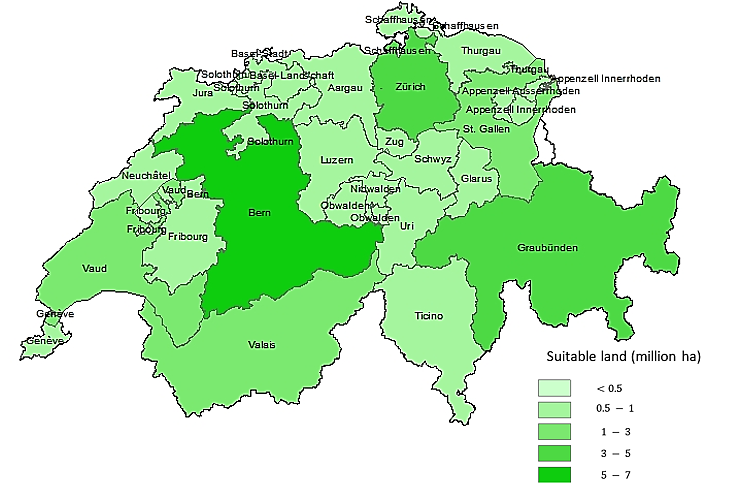


Fig. 4. Map of suitable land in Switzerland: total area in each Canton

- 1. **Waste heat supplier analysis**

Municipal solid waste incinerators, cement production plants and biogas plants have a combined waste heat potential of ~300 MW (Fig. 5). Although the largest number of waste heat suppliers are biogas plants (81%), their waste heat potential contributes the smallest (10%). On the contrary, municipal solid waste incinerators (MSWI) have the highest waste heat potential percentage (60%) while they account for only 16% of the total supplier’s number.

As Fig. 6 demonstrates, most biogas plants have less than 1 MW waste heat potential. Most municipal solid waste incinerators (MSWI) have a waste heat potential of 1 to 10 MW. Finally, the waste heat potential of cement production plants is more than 10 MW.


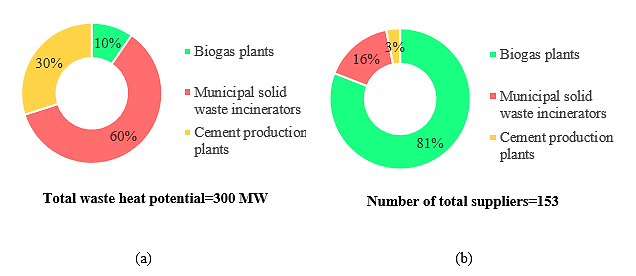


Fig. 5. Quantity and waste heat potential of selected waste heat suppliers (biogas plants, MSWI and cement production plants) in Switzerland, (a) Waste heat potential of each category, (b) Number of suppliers based on their category


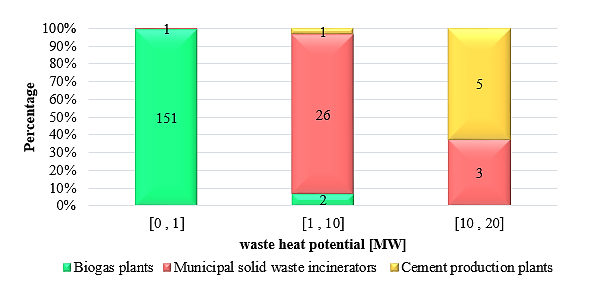


Fig. 6. Contribution of selected waste heat suppliers (biogas plants, MSWI and cement production plants) regarding their waste heat potential in Switzerland

- 1. **. Economy of scale**

The effect of the economy of scale (decreasing the unit investment cost by increasing the numbers or capacity) on the optimization results is depicted in Fig. 7. In this figure, the optimal points with and without considering the economy of scale effect are compared. As can be seen, without the investment cost adjustment, the optimum points are sparsely distributed (151 hotspots), while considering the economy of scale, the number of optimal points will be decreased (95 hotspots). Therefore, the economy of scale can considerably influence the optimization results moving towards large-scale greenhouses. Hereby, the total costs decreased by 37%.


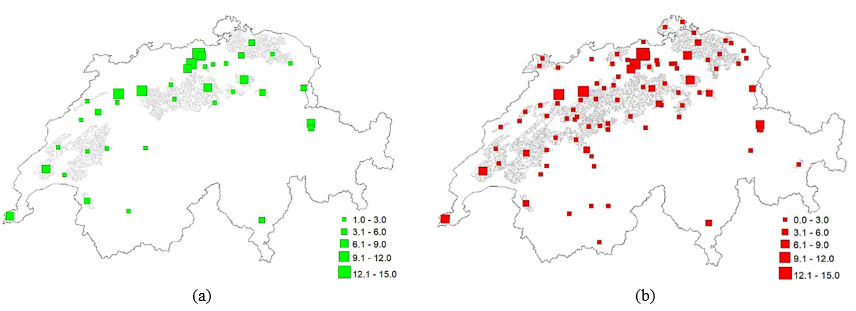


Fig. 7. The effect of considering economy of scale on the optimum results (100 ha), (a) Optimum answers considering the economy of scale (ha), (b) Optimum answers without economy of scale consideration (ha).

- 1. **ORC**

Due to the high investment cost of ORC technology, the direct heat transfer (pipeline) is selected as optimal pathway for all scenarios. Increasing electricity prices by a factor of 3 to ~0.5 CHF/kWh would change the results: ORC would be chosen by the optimization model for 100-hectare greenhouse area (Fig. 8). The objective function, in this case, declines to negative values, which means this installation will be profitable in this condition. Only cement production plants are selected for installing ORC technology. The reason is that the cement production plants have considerable waste heat potential, and since the economy of scale for ORC investment cost is considered, the cement production plants are highlighted as the optimal suppliers’ points for ORC technology implementation. Therefore, in this new situation, greenhouses use the rejected heat of the ORC condenser (supplied by the waste heat of cement production plants), while electricity is also generated (totally about 5 MW) in this process.


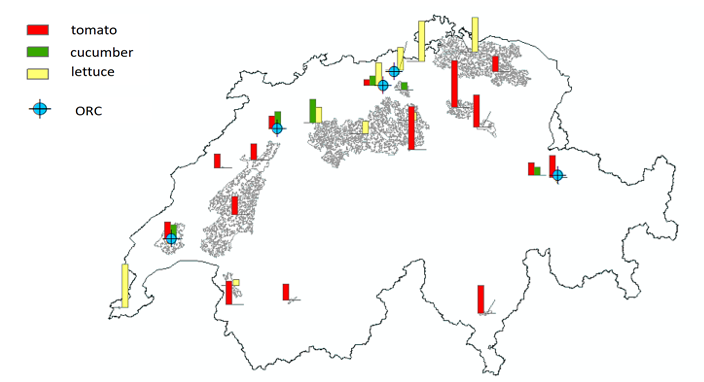


Fig. 8. The location of greenhouses and ORC systems with tripled electricity price (ha)

- 1. **Contribution analysis of objective function terms**

Fig. 9 shows the contribution of the various cost terms of the objective function. The investment cost varies between 70-90% of the total annualized cost. The investment cost of the pipeline rises as the greenhouse area expands from 20 ha to 195 ha. Note that as the target greenhouse area grows, the distance between the greenhouses and waste heat suppliers also increases. So the model incorporates a longer distance to meet the demand, which results in an increase in the associated cost.

It should be noted that in this optimization model, five specific ORC capacities (1 MW, 2MW, 3MW, 4MW and 5MW) are considered as the feasible options for electricity generation. However, due to high investment cost of ORC and the electricity selling price (see section 3.4. ORC), the ORC technology is not included in the optimal solution.


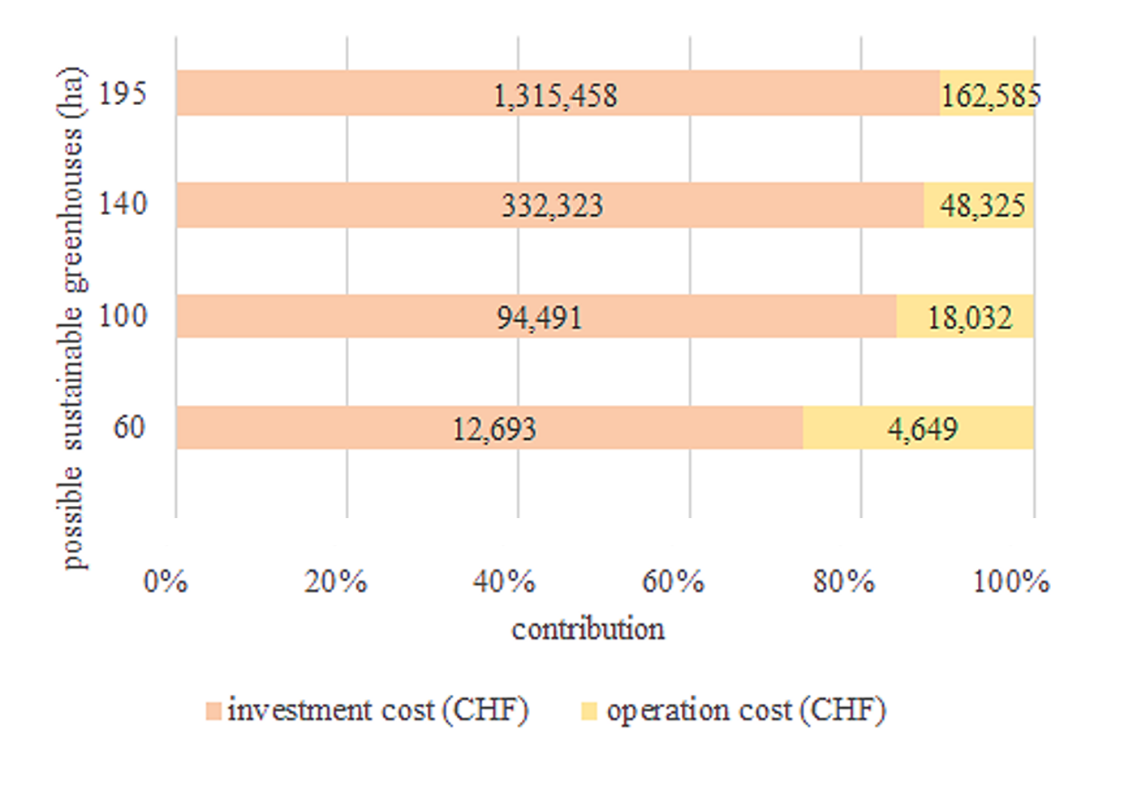


Fig. 9. The contribution of fixed (pipeline) and variable (electricity for pumping warm water) costs for various levels of greenhouse area implementations (in ha). Note that the investment cost of heat exchanger in waste heat recovery system of waste heat suppliers should be added to the total cost of industrial symbiosis network.

### References

1. Zuberi, M. J. S., & Patel, M. K. (2017). Bottom-up analysis of energy efficiency improvement and CO2 emission reduction potentials in the Swiss cement industry. Journal of Cleaner Production, 142, 4294–4309. <https://doi.org/10.1016/j.jclepro.2016.11.178>
2. CORDIS, cordis.europa.eu. (2022, August 17). Waste Heat Recovery for Power Valorisation with Organic Rankine Cycle Technology in Energy Intensive Industries. CORDIS | European Commission. https://cordis.europa.eu/project/id/637189/results
3. Einheitliche Heizwert- und Energiekennzahlenberechnung der Schweizer KVA nach europäischem Standardverfahren (2020).
4. Heat, C. (2021). Power-Technologies, A detailed guide for CHP developers, Department for Business. Energy and Industrial Strategy.
5. Dokumentation Geodatenmodell, Biogasanlagen (2022).
6. Scholwin, F., & Nelles, M. (2013). Energy flows in biogas plants: analysis and implications for plant design. 212–227. <https://doi.org/10.1533/9780857097415.2.212>
7. Nussbaumer, T., Thalmann, S., Jenni, A., & Ködel, J. (2020). Handbook on planning of district heating networks. Bern: Swiss Federal Office of Energy.
8. Zhang, Chuan, et al. "A novel methodology for the design of waste heat recovery network in eco-industrial park using techno-economic analysis and multi-objective optimization." Applied energy 184 (2016): 88-102.
9. Zuberi, M. J. S., Bless, F., Chambers, J., Arpagaus, C., Bertsch, S. S., & Patel, M. K. (2018). Excess heat recovery: An invisible energy resource for the Swiss industry sector. Applied Energy, 228, 390–408. https://doi.org/10.1016/j.apenergy.2018.06.070
10. Wang, K., Simpson, M. L., Rotolo, G., Distaso, E., Markides, C. N., Sapin, P., & P. De Palma. (2019). Thermoeconomic optimisation of small-scale organic Rankine cycle systems based on screw vs. piston expander maps in waste heat recovery applications. Energy Conversion and Management, 200, 112053–112053. https://doi.org/10.1016/j.enconman.2019.112053
11. Valencia, G., Fontalvo, A., & Duarte Forero, J. (2021). Optimization of waste heat recovery in internal combustion engine using a dual-loop organic Rankine cycle: Thermo-economic and environmental footprint analysis. Applied Thermal Engineering, 182, 116109. https://doi.org/10.1016/j.applthermaleng.2020.116109
12. Vivian, J., Manente, G., & Lazzaretto, A. (2015). A general framework to select working fluid and configuration of ORCs for low-to-medium temperature heat sources. Applied Energy, 156, 727–746. https://doi.org/10.1016/j.apenergy.2015.07.005
13. Copernicus, Global Monitoring for Environment and Security, the European Union's Earth observation programme| Copernicus. (n.d.). Www.copernicus.eu. Retrieved July 25, 2023, from http://www.copernicus.eu/en
