# Supplementary material for: Spatial optimization of industrial symbiosis for heat supply of agricultural greenhouses
Source: J Ind Ecol. 2024 Aug 13;28(6):1507–23. doi: 10.1111/jiec.13543 (PMC11667671; doi:10.1111/jiec.13543)

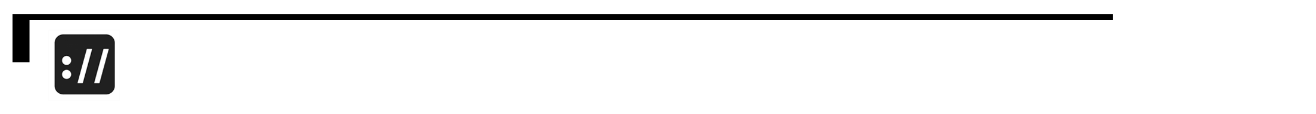


SUPPORTING INFORMATION FOR:

Rezaei, F., Burg, V. , Pfister, S. , Hellweg, S. & Roshandel, R. (2024.) Spatial Optimization of Industrial Symbiosis for Heat Supply of Agricultural Greenhouses. *Journal of Industrial Ecology.*


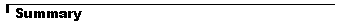


Supporting information S2: This Supporting Information provides the locations and practical waste heat potential of selected suppliers in Switzerland (Appendix SI-4); peak heat demand for suitable lands for tomato, cucumber, and lettuce greenhouses in Switzerland (Appendix SI-5); opportunity map for replacing imported vegetables (tomato, cucumber, and lettuce) with domestic greenhouse crops (Appendix SI-6); locations of possible greenhouses and vegetables for 20 hectares, 60 hectares, 100 hectares and 140 hectares scenarios (Appendix SI-7); effect of targeted greenhouse area including 20, 60, 100, and 140 ha on the objective function value and total utilized waste heat in optimum points and waste heat source contributions (Appendix SI-8).


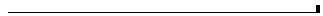

Supplement: Supplementary file 2 — Supporting Information S2: Also, Supporting Information S2 provides the locations and practical waste heat potential of selected suppliers in Switzerland (Appendix SI-4); peak heat demand for suitable lands for tomato, cucumber, and lettuce greenhouses in Switzerland (Appendix SI-5); opportunity map for replacing imported vegetables (tomato, cucumber, and lettuce) with domestic greenhouse crops (Appendix SI-6); locations of possible greenhouses and vegetables for 20 hectares, 60 hectares, 100 hectares and 140 hectares scenarios (Appendix SI-7); effect of targeted greenhouse area including 20, 60, 100, and 140 ha on the objective function value and total utilized waste heat in optimum points and waste heat source contributions (Appendix SI-8). [file 44498_2024_2806014_MOESM2_ESM.zip › read me!.docx]
